# Regulation of the RNA and DNA nuclease activities required for *Pyrococcus furiosus* Type III-B CRISPR–Cas immunity

**DOI:** 10.1093/nar/gkaa176

**Published:** 2020-03-21

**Authors:** Kawanda Foster, Sabine Grüschow, Scott Bailey, Malcolm F White, Michael P Terns

**Affiliations:** 1 Department of Microbiology, University of Georgia, Athens, GA 30602, USA; 2 Biomedical Sciences Research Complex, School of Biology, University of St Andrews, St Andrews KY16 9ST, UK; 3 Department of Biochemistry and Molecular Biology, Bloomberg School of Public Health, Johns Hopkins University, Baltimore, MD 21205, USA; 4 Department of Biochemistry and Molecular Biology, University of Georgia, Athens, GA 30602, USA; 5 Department of Genetics, University of Georgia, Athens, GA 30602, USA

## Abstract

Type III CRISPR–Cas prokaryotic immune systems provide anti-viral and anti-plasmid immunity via a dual mechanism of RNA and DNA destruction. Upon target RNA interaction, Type III crRNP effector complexes become activated to cleave both target RNA (via Cas7) and target DNA (via Cas10). Moreover, trans-acting endoribonucleases, Csx1 or Csm6, can promote the Type III immune response by destroying both invader and host RNAs. Here, we characterize how the RNase and DNase activities associated with Type III-B immunity in *Pyrococcus furiosus* (*Pfu*) are regulated by target RNA features and second messenger signaling events. *In vivo* mutational analyses reveal that either the DNase activity of Cas10 or the RNase activity of Csx1 can effectively direct successful anti-plasmid immunity. Biochemical analyses confirmed that the Cas10 Palm domains convert ATP into cyclic oligoadenylate (cOA) compounds that activate the ribonuclease activity of *Pfu* Csx1. Furthermore, we show that the HEPN domain of the adenosine-specific endoribonuclease, *Pfu* Csx1, degrades cOA signaling molecules to provide an auto-inhibitory off-switch of Csx1 activation. Activation of both the DNase and cOA generation activities require target RNA binding and recognition of distinct target RNA 3′ protospacer flanking sequences. Our results highlight the complex regulatory mechanisms controlling Type III CRISPR immunity.

## INTRODUCTION

Prokaryotes often harbor powerful CRISPR–Cas (clustered regularly interspaced short palindromic repeat-CRISPR associated) adaptive immune systems to protect against infections from invading viruses and plasmids (1,2). CRISPR genomic arrays are composed of short DNA sequences of foreign origin (called spacers), separated by host repeat sequences. CRISPR arrays become transcribed and the long, primary transcripts are processed into short, mature crRNAs that assemble with Cas proteins to form crRNP (CRISPR RNA-containing ribonucleoprotein) effector complexes. These effector complexes detect and destroy invading nucleic acids that are complementary to their crRNAs. CRISPR–Cas systems are quite diverse and fall into six distinct types (Types I-VI) and over 30 subtypes (3,4). Types I, II and V (and possibly IV) target the destruction of DNA (5–7), while Type VI destroys RNA (8). Type III systems are particularly noteworthy in that they uniquely degrade both RNA and DNA of the invaders (9–19). Type III systems are further categorized into six subtypes (III-A through III-F) with the majority belonging to either the Type III-A (Csm) or Type III-B (Cmr) systems (3).

**Figure 1.**
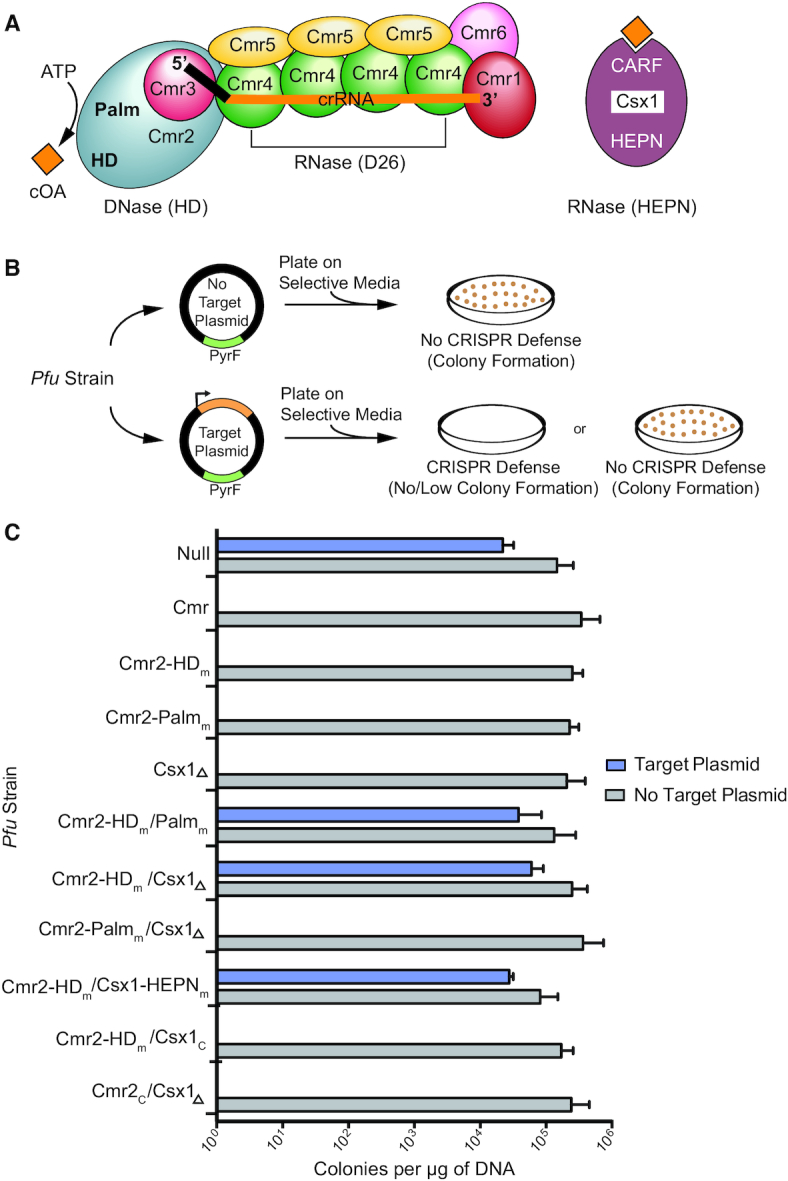
Csx1 is required for anti-plasmid immunity in the *P. furiosus (Pfu)* III-B CRISPR–Cas system. (**A**) Components of the *Pfu* Cmr defense response. The Cmr effector complex is composed of Cmr1–6 and a mature crRNA (black & orange) containing a 5′ tag (black) eight nucleotides in length. Cyclic oligoadenylate compounds (orange) produced by the Palm domain of Cmr2 bind to the CARF domain of Csx1. See Supplemental Figure S1 for a list of the Cas gene family names that correspond to each of the six Cmr subunits. (**B**) Plasmid interference assay. *Pfu* strains were transformed with plasmids that contain (target) or lack (no target) a transcribed target region complementary to the 7.01 crRNA (first spacer of the *Pfu* CRISPR 7 array). Both plasmids contain the *pyrF* gene to facilitate growth in the absence of uracil. (**C**) Colonies produced by transforming 11 different *Pfu* strains. Strains were transformed with the target plasmid (blue) or no target plasmid (gray). Mean colonies obtained per μg of plasmid DNA are plotted for two replicates. Error bars indicate standard deviation of replicates.

Types III-A (Csm) and III-B crRNP (Cmr) effector complexes exhibit an overall similar subunit organization and architecture (see Figure 1A for an example of the Cmr effector complex). Each complex is composed of a single crRNA and five (Csm 1–5 for III-A) or six (Cmr 1–6 for III-B) Cas proteins (15,20–25). The mature crRNAs within these complexes contain eight nucleotides of repeat sequence at the 5′ end called the 5′ tag, followed by a ∼30–40 nucleotide guide sequence that base-pairs with the target RNA protospacer (26–28). Multiple catalytically active Cas7 superfamily proteins (Csm3 or Cmr4) that act as target RNA endoribonucleases (16,29,30), interact along the length of the crRNA guide region and these proteins also tightly associate with Cas11 superfamily proteins (Csm2 or Cmr5). Additional Cas7 superfamily Cas proteins directly contact the 5′ crRNA tag (Csm4 or Cmr3) or 3′ terminus of the guide RNA segment (Csm5 or Cmr1 and Cmr6). Cas10 (Csm1 or Cmr2) is the signature protein of Type III complexes (4). This large, multiple domain-containing protein is situated near the 5′ end of the crRNA and typically contains two highly conserved motifs: the HD motif capable of destroying single-stranded DNA (9,10,13) and the GGDD motif of one of two Palm domains that can convert ATP into cyclic oligoadenylate (cOA) second messenger molecules (31–33) (Figure 1A).

Interestingly, Type III systems also include trans-acting ribonucleases, Csm6 (III-A) or Csx1 (III-B), that are not stably associated with the effector crRNP complexes (34–37) and appear to be capable of degrading both invading RNA (leading to immunity) as well as host RNAs (leading to cell dormancy or cell death) when activated by cOA binding (32,35,38). Csm6 and Csx1 proteins share two highly conserved domains: the HEPN (Higher Eukaryotes and Prokaryotes Nucleotide binding) domain and the CARF (CRISPR Associated Rossman Fold) domain (39,40) (Figure 1A). The ribonuclease activity of both Csx1 and Csm6 is facilitated by the HEPN motif (R-X_4–6_-H) within the HEPN domain (34–37). Binding of cognate cOA to the CARF domain allosterically stimulates the single-stranded RNase activity of the HEPN domain of the Csm6/Csx1 proteins (31–33,41,42). CARF domains, either within Csm6 (43,44) or as part of unrelated proteins called ring nucleases (45,46), have also recently been found to cleave and inactivate cOA singling molecules to switch off the activity of the Csm6 or Csx1 HEPN RNases (43–46).

Anti-virus or anti-plasmid immunity afforded by Type III crRNPs, is transcription-dependent as these systems specifically recognize an RNA target having a crRNA interaction region (i.e. the RNA protospacer) (9,10,14,19,47). Once bound to the target RNA protospacer, the crRNP effector complex changes its conformation (24,25,48–50) and the Type III defense response becomes activated to: (i) specifically cleave the target RNA at regular six-nucleotide intervals within the protospacer region using multiple copies of the Csm3/Cmr4 integral ribonuclease via an active site that relies on a key aspartate residue (16,29,30), (ii) non-specifically degrade nearby invading single-stranded DNA via the HD motif of Cas10 (Csm1/Cmr2) (9,10,13,14), (iii) generate cyclic oligoadenylate (cOA) from ATP using the GGDD motif of the conserved Palm domain of Cas10 (Csm1/Cmr2) and (iv) non-specifically degrade single-stranded RNA via the cOA-activated and trans-acting Csm6/Csx1 HEPN ribonuclease (31–33,41,42,51,52). Collectively, these target-RNA and transcription-coupled reactions provide robust Type III-mediated immunity.

Type III RNase and DNase activities must be tightly regulated so that undesirable cellular toxicity or host cell death can be prevented before or during an immune response. The molecular details for how each of the RNases, DNase and cOA signal generation activities of Type III systems become specifically activated by target RNA binding is currently not fully understood. Several studies have revealed a key role for the short sequence that flanks the 3′ end of the RNA protospacer, termed the protospacer flanking sequences (PFS) in controlling Type III activities. When PFS sequences are complementary to the 5′ crRNA tag (as would be the case if the cell produced antisense crRNAs), the DNase and cOA production activities fail to become activated (9,13,14,53,54) while the transcripts themselves are cleaved by the Csm3/Cmr4 integral RNases (16,27). Thus, 5′ tag/PFS pairing can negatively regulate some but not all Type III activities. In some characterized systems, the particular identity of nucleotides within the PFS will dictate if the effector complex is active or inactive independent of their capacity to base-pair with the 5′ crRNA tag. In particular, three nucleotides immediately 3′ of the target RNA protospacer (i.e. in positions +1, +2, +3 in the target RNA 5′-3′ direction) have been found to be important for activating the DNase and cOA production presumably due to interactions of these PFS elements and subunits of the Type III effector complex (likely Cas10 and/or Csm4/Cmr3) (9,25,48,54). The DNase and cOA generation activities are also switched off as a result of degradation of the trigger target RNA that occurs when Csm3/Cmr4 RNases of the complex cleave the RNA protospacer (33,42,48). Finally, mechanisms have also recently been discovered that can reverse the effects of cOA signaling on Csm6/Csx1 HEPN ribonuclease activity. Specifically, dedicated ‘ring nucleases’ have been identified that bind, cleave, and inactivate cOAs by converting the compounds into short, bi-adenylate degradation products with 2′-3′ cyclic phosphate termini (A_2_ > p) in certain organisms (45,46). However, other organisms appear not to possess distinct ring nucleases and instead have been found to rely on an intrinsic ability of the CARF domains of Csm6/Csx1 to destroy the cOA molecules (43,44). Additional layers and molecular mechanisms for controlling the activity of each of the key nucleases of Type III effector crRNPs as well as the cOA signaling pathway likely await discovery.

*Pyrococcus furiosus* (*Pfu*) is a hyperthermophilic archaeon that contains three distinct functional CRISPR–Cas effector crRNPs: two DNA-targeting systems (Types I-A (Csa) and I-B (formerly known as I-G) (Cst) (55–59)) and a Type III-B (Cmr) system (Supplemental Figure S1) shown to destroy both DNA and RNA targets in a transcription-coupled manner (9,11,17,27). Previous investigations on the mechanism of action of *Pfu* Cmr crRNPs also revealed that crRNA-mediated target RNA interaction was required for both target RNA cleavage (by Cmr4) (11,17,29) and non-specific DNase activity (by HD domain of Cmr2) which was shown to cleave single-stranded DNA substrates or both strands of short double-stranded DNA substrates (9). The target RNA PFS requirements for controlling immunity by *Pfu* Cmr crRNPs have been thoroughly investigated. Similar to other studied Type III systems, PFS sequences capable of base-pairing with the 5′ tag sequence of the crRNA prevented DNase activity but not target RNA destruction (9,11). In contrast, the identity of three nucleotides within the PFS immediately 3′ of the target RNA protospacer (previously referred to as the rPAM [RNA protospacer-adjacent motif] (9)), was critical for DNase activity *in vitro* and anti-plasmid immunity *in vivo*, but not target RNA cleavage (9). Whether or not *Pfu* crRNPs are capable of producing cOA second messenger compounds had not yet been tested. Furthermore, deletion of the *csx1* gene in *Pfu* did not disrupt anti-plasmid immunity (9). These findings raised the key question as to whether cOA-mediated activation of Csx1 RNase activity is important for conferring immunity by the *Pfu* Cmr crRNP effector complex. In this study, we expand our understanding of the molecular mechanisms of action of the well-characterized Type III-B (Cmr) system of *Pyrococcus furiosus* (*Pfu*) and further define how the Csx1 RNase and Cmr2 DNase activities are regulated by target RNA elements and second messenger signaling events.

## MATERIALS AND METHODS

### *P. furiosus* strains and growth conditions

All *P. furiosus* strains utilized in this study are listed in Supplemental Table S1. Strains were grown at 90°C under strict anaerobic conditions using defined medium as previously detailed (60). Cultures were grown in 5 or 20 mL volumes and inoculated with either 1% inoculum or a single isolated colony. Cultures were incubated for 16–24 h and plates were incubated for 64 h. Uracil (20 μM) and/or 5-fluoroorotic acid (5-FOA, 2.75 mM) were supplemented in the media for selection or counterselection for the *pyrF* marker gene.

*P. furiosus* strains were produced using homologous recombination of transformed SOE-PCR (splicing by overlap extension-polymerase chain reaction) constructs as previously reported (9). Complementation strains were generated by adding a modified wildtype *cmr2* or *csx1* gene back onto the *P. furiosus* genome that contained restriction sites that enabled detection of the introduced genes from wildtype genes and did not affect the protein coding potential. A BclI-HF (5′-TGATAA-3′ → 5′-TGATCA-3′) restriction site was introduced into *cmr2* and a NruI-HF (5′-TCGGGA-3′ → 5′-TCGCGA-3′) restriction site was introduced into *csx1*. Primers used to make the complementation strains can be found in Supplemental Table S3. All strains underwent at least three rounds of strain purification using minus uracil selective media. Successful strain generation was confirmed via PCR amplification of the *Pfu* gene of interest and DNA sequencing (Eurofins Genomics).

### Recombinant protein expression and purification

The genes encoding *P. furiosus* Cmr1–6 and Csx1 proteins were amplified via PCR and cloned into modified versions of pET24-D (Cmr4, Cmr5, Csx1), pET101-D (Cmr1–1) and pET200-D (Cmr2, Cmr3, Cmr6) as previously detailed (17,37). All constructs contain a 6x-histidine tag on either the N-terminus (Cmr2–6, Csx1) or C-terminus (Cmr1–1) of the corresponding protein. Recombinant protein expressions were performed in *E. coli* BL21-RIPL cells (DE3, Novagen). Expression cultures for wildtype and mutant proteins were grown in 1 l (Cmr1–1, Cmr4, Cmr5, Csx1), 2 l (Cmr2, Cmr3) or 4 l (Cmr6) cultures at 37°C. Luria broth (Cmr1–1, Cmr2–Cmr5, Csx1; Research Products International (RPI)) or Terrific broth (Cmr6; RPI) medium was used for cultures and supplemented with either 50 μg/ml kanamycin sulfate (Cmr2-Cmr6, Csx1) or 100 μg/ml ampicillin (Cmr1–1) for plasmid selection. Cultures were grown to an OD_600_ of 0.7 at 37°C then induced with 0.5 mM *iso*-propyl-$\beta$-d-thiogalactopyranoside (IPTG) at 24°C overnight. Cells were pelleted then resuspended in lysis buffer (40 mM Tris–HCl (pH 7.5), 500 mM NaCl, 10 mM Imidazole) containing one protease inhibitor tablet (Roche) and lysed via sonication. Cell lysates underwent a thermal precipitation by incubating in a 75°C bead bath for 20 minutes. Insoluble material was removed by centrifugation at 14,000 rpm for 20 minutes at 4°C and filtered through a 0.8 μM syringe filter (Corning Incorporated). Proteins were purified using gravity affinity chromatography and either Ni-NTA resin (Cmr2, Cmr3, Cmr4, Cmr5, Cmr6, Csx1; Thermo Scientific) or Talon Cobalt resin (Cmr1–1; Clontech). Cell lysates were rotated with pre-rinsed and equilibrated resin for 1 h at 4°C. The proteins were then washed with the lysis buffer and wash buffer (40 mM Tris–HCl (pH 7.5), 500 mM NaCl, 20 mM Imidazole). The proteins were then eluted using four different elution buffers containing increasing amounts of imidazole (50, 100, 200, 500 mM). Wildtype and HEPN mutant Csx1 proteins were dialyzed and underwent a second round of gravity affinity chromatography. Buffer exchange was performed using Slide-A-Lyzer Dialysis Cassettes (Thermo Scientific) in elution buffer lacking Imidazole. Protein concentrations were assessed using Qubit protein concentration assays (Invitrogen) and purity was assessed via SDS-PAGE and Coomassie blue staining analysis.

### Csx1 mutagenesis

The wildtype gene encoding Csx1 was subcloned into a modified pET24D vector. The Csx1 HEPN mutant (Csx1-HEPN_m_) contains a H436A mutation and was created as previously described (37). CARF domain mutants were created using the wildtype vector and either inverse PCR or Quikchange site-directed mutagenesis (Stratagene). Mutagenesis primers are provided in Supplemental Table S3. Amino acid residues 121–127 were deleted from Csx1 using inverse PCR to create a mutant form of the protein predicted to not be able to organize a functional CARF motif (Csx1-CARF_m_). Quikchange mutagenesis was used to create site-specific mutations within the predicted cOA binding pocket of the CARF domain of *Pfu* Csx1. Two cOA binding mutants were created: Csx1-INAA (I169A, N170A) and Csx1-INQQ (I169Q, N170Q). Successful mutagenesis for all mutant plasmids was confirmed via DNA sequencing (Eurofins Genomics).

Purification of Csx1-HEPN_m_ was performed as described above. Purification of wildtype, Csx1-CARF_m_, Csx1-INAA and Csx1-INQQ proteins was performed using a batch method. The batch method involved affinity purifying the proteins by incubating the soluble lysate with Ni-NTA resin for 1 h at 4°C then performing subsequent washes and elutions in a 15 ml centrifuge tube and spinning at 4000 rpm for 2 mins in between each step. Protein concentrations were assessed using Qubit assays and purity was assessed via SDS-PAGE and Coomassie blue staining analysis.

### Preparation of RNA and DNA substrates

Synthetic RNAs (7.01 crRNA and 7.01 Target RNA) were purchased from Integrated DNA Technologies and DNAs from Eurofins Genomics. The RNA and DNA sequences can be found in Supplemental Table S3. Synthetic target RNA was 5′-end labeled using γ-^32^P-ATP, gel purified, eluted, extracted, and precipitated as previously described (37). 7.01 crRNA was gel purified, eluted, extracted, and precipitated prior to using in assays. 5′-end labeled, double-stranded DNA substrate was prepared by annealing complementary DNA oligonucleotides as previously described (37) and gel purification.

Target RNAs with the indicated PFS sequences were created by *in vitro* transcription using T7 RNA polymerase and the MEGAshortscript T7 kit (Invitrogen) as described (9). DNA templates with a T7 phage promoter sequence were generated by amplifying a target plasmid listed in Supplemental Table S2 with IVT primers listed in Supplemental Table S3 and gel purified using the Zymoclean Gel Recovery Kit (Zymo Research). Following synthesis, target RNAs were subsequently gel purified from denaturing gels, eluted, extracted, and precipitated prior to adding to the assays. Target RNA concentrations were determined using the Qubit RNA BR Assay Kit (Invitrogen) and quality was assessed using 7M urea denaturing 15% polyacrylamide gels and ethidium bromide staining (Supplemental Figure S2).

### Plasmid interference assay

Plasmid transformation interference assays were performed as previously described (9). Liquid cultures of *P. furiosus* strains were allowed to reach mid-to-late log phase of growth. 100 μl of liquid culture was transformed with 1 ng of either Target plasmid (pJE65; containing a transcribed protospacer matching the 7.01 crRNA) or No Target (pJE47) control plasmid. The transformations were incubated for 15–45 min at room temperature prior to plating. Each transformation mixture was split between two plates and spread onto solid defined media lacking uracil. The plates were incubated at 90°C in an anaerobic chamber. Plates were observed for colony growth and counted after 64 h of incubation. Results shown represent two replicates.

### Cmr crRNP *in vitro* activity assays

RNA and DNA nuclease activity assays were performed similarly to methods previously described (9,17). Purified recombinant Cmr proteins were first incubated with 7.01 crRNA to form crRNPs. For RNase assays, Cmr crRNPs were assembled by preincubating 500 nM of each Cmr protein (50 nM of Cmr2) with RNA assay buffer (20 mM Tris–HCl (pH 7.5), 250 mM NaCl, 1.5 mM MgCl_2_), and 12.5 nM of 7.01 crRNA for 25 min at 70°C. After the preincubation, 0.5–1.5 nM of radiolabeled synthetic 7.01 target RNA was added to the reaction and incubated for one hour at 70°C. One unit of Proteinase K (NEB) was then added to each reaction and incubated for 30 min at 37°C prior to gel electrophoresis. For DNase assays, the Cmr crRNP was assembled in DNA assay buffer (20 mM Tris–HCl (pH 7.5), 250 mM NaCl, 1.5 mM MgCl_2_, 200 μM NiCl_2_) and 50 nM of 7.01 crRNA for 25 min at 70°C. After the preincubation, 100 nM of 7.01 target RNA and 1 nM of radiolabeled dsDNA was added to the reaction and incubated for one hour at 70°C. Unless otherwise indicated, DNase assays were completed with target RNA containing a 5′-GGG-3′ PFS sequence. One unit of Proteinase K was then added to each reaction and incubated for 30 min at 37°C prior to gel electrophoresis. Cyclic oligoadenylate production assays were performed by assembling the Cmr crRNPs as described above using RNA assay buffer and 50 nM of 7.01 crRNA. After assembly, 0.5 mM of ATP (NEB), 5 nM of α-^32^P-ATP (3000 Ci/mmol; Perkin Elmer), and 100 nM of target RNA was added to the reaction and incubated for 1 h at 70°C. Unless otherwise indicated, cOA production assays were completed with target RNA containing a 5′-GGG-3′ PFS sequence. All Reactions were stopped by adding Gel Loading Buffer II (Life Technologies) and visualized by using 7M urea denaturing 15% polyacrylamide gels followed by autoradiography. Cyclic oligoadenylate reactions were also ran on 8M urea denaturing 20% polyacrylamide sequencing gels. Decade Markers (Life Technologies) and partial alkaline hydrolysis ladders (Ambion) of poly A_19_ RNA were generated as previously described (34,37).

### Csx1 *In vitro* activation assays

#### Activation with the native Cmr produced cOA

Csx1 RNase activity assays were performed as previously reported (37). Ribonuclease activity of Csx1 was assessed by incubating 500 nM of Csx1 with 0.5–1.5 nM of radiolabeled target RNA in assay buffer (20 mM Tris–HCl (pH 7.5), 200 mM NaCl) for 1 h at 70°C. In order to assess activation of Csx1, 10 or 20 nM of Csx1 was incubated with radiolabeled target RNA and assay buffer in the presence of unlabeled cOA for 1 h 70°C. Unlabeled native cOA was generated as described above by omitting α-^32^P-ATP. The unlabeled cOA was then extracted similarly to published methods (61). Five reaction volumes of phenol/chloroform/isoamyl alcohol (PCI, 125:24:1 at pH 4.5; Ambion) was added to the reaction and vortexed for 30 seconds. The mixture was then centrifuged at 20 000 rpm at 4°C then the aqueous layer incubated with five reaction volumes of chloroform (Fisher Scientific) vortexed and centrifuged. The aqueous layer was extracted, aliquoted, and stored at −80°C in single use aliquots. All reactions were stopped by adding Gel Loading Buffer II and visualized by using 7M urea denaturing 15% polyacrylamide gels followed by autoradiography.

#### Activation with synthetic cA_4_ and cA_6_

Csx1 ribonuclease activation assays with cA_4_ and cA_6_ species was performed as described above except synthetic cOAs from BIOLOG Life Science Institute were used. Several concentrations of cA_4_ and cA_6_ were tested as indicated in the figure legend.

#### Activation with Csx1-treated cOA

Cmr crRNP-generated cOA was incubated with or without 600 nM of wildtype Csx1 for 30 min at 70°C. Reaction products were then extracted to deproteinize the samples (as described above) and incubated with 20 nM of Csx1, 5′-end labeled target RNA, and assay buffer for 1 h at 70°C.

### Mass spectrometry

Unlabeled cOA production assays were performed as described above except the reactions were incubated for 2 h. Liquid chromatography high resolution mass spectrometry (LC-HRMS) analysis was performed on a Thermo Scientific Velos Pro instrument equipped with HESI source and Dionex UltiMate 3000 chromatography system as previously described (41).

## RESULTS

### Effective anti-plasmid immunity is achieved by either Csx1 RNase or Cas10 DNase activity

Distinct Type III-A or III-B systems have shown considerable variability in the need for Cas10 (Csm1/Cmr2) DNase and/or Csm6/Csx1 RNase activities for anti-plasmid and anti-viral immunity (9,32,34,35,38,47,62). In our earlier *in vivo* work with the *Pfu* Cmr (III-B) system, we found that individual mutations of either the HD (H13A/D14A) or Palm domain GGDD (D673A/D674A) motifs of Cmr2 (Cmr2-HD_m_ or Cmr2-Palm_m_ mutants) or a single deletion of the *csx1* gene (Csx1_Δ_), did not interfere with anti-plasmid immunity but double mutations in the Cmr2 HD and GGDD Palm motifs (Cmr2-HD_m_/Palm_m_) prevented immunity (9) (see Figure 1A for overview of the crRNA and Cas protein components). This early work was performed prior to knowledge that the Palm domain of some Cas10 superfamily proteins can catalyze conversion of ATP to cOA signaling molecules that activate the ribonuclease activity (HEPN domain) of Csm6/Csx1 HEPN ribonucleases (31–33,41,42,51,52). This new information motivated us to perform a more systematic *in vivo* mutational analyses in which specific combinations of double mutants were tested to more fully address if the Cmr2 DNase and Csx1 RNase activities were important for anti-plasmid immunity (Figure 1).

Anti-plasmid immunity was assayed *in vivo* by transforming a *Pfu* strain containing wildtype or mutant versions of the Cmr system with a target plasmid that harbors a transcribed protospacer matching an endogenous crRNA (7.01; the first crRNA from CRISPR locus 7) or empty plasmid control (Figure 1B). As we observed previously, the anti-plasmid immunity observed with wildtype Cmr was unaffected when Cmr effector complexes contained mutations in either the Cmr2 DNase HD active site (Cmr2-HD_m_) or Palm GGDD motif (Cmr2-Palm_m_), or if the *csx1* gene was deleted from the genome (Csx1_Δ_) (Figure 1C and (9)). As expected, immunity was absent for a strain that lacked the entire Cmr complex and Csx1 (null strain) or when the Cmr2-HD_m_/Palm_m_ double mutant was re-tested. Immunity was disrupted when the *Pfu* csx1 gene was deleted or contained a mutation within the RNase catalytic motif of *csx1* (Csx1-HEPN_m_; H436A) in conjunction with a second mutation within the DNase catalytic site of Cmr2 (Cmr2-HD mutation) (Figure 1C and (9)). These same *csx1* mutations did not prevent immunity when combined with a Cmr2-Palm mutation (GGDD; predicted to block cOA generation). Cmr-mediated immunity was rescued by restoring either wildtype *cmr2* or *csx1* genes in the Cmr2-HD_m_/Csx1_Δ_ double mutant strains (Cmr2c and Csx1c are Cmr2-HD_m_/Csx1_Δ_ strains complemented with wt *cmr2* or wt *csx1*, respectively. Figure 1C). Taken together, the results show that both the DNase activity of the Cmr effector crRNPs (via HD domain of Cmr2) as well as the RNase activity of Csx1 (via HEPN motif and activated by Cmr2-Palm GGDD motif) are each sufficient for conferring highly effective anti-plasmid immunity in *Pfu*.

### *P. furiosus* Cmr crRNPs produce cyclic oligoadenylate second messengers

**Figure 2.**
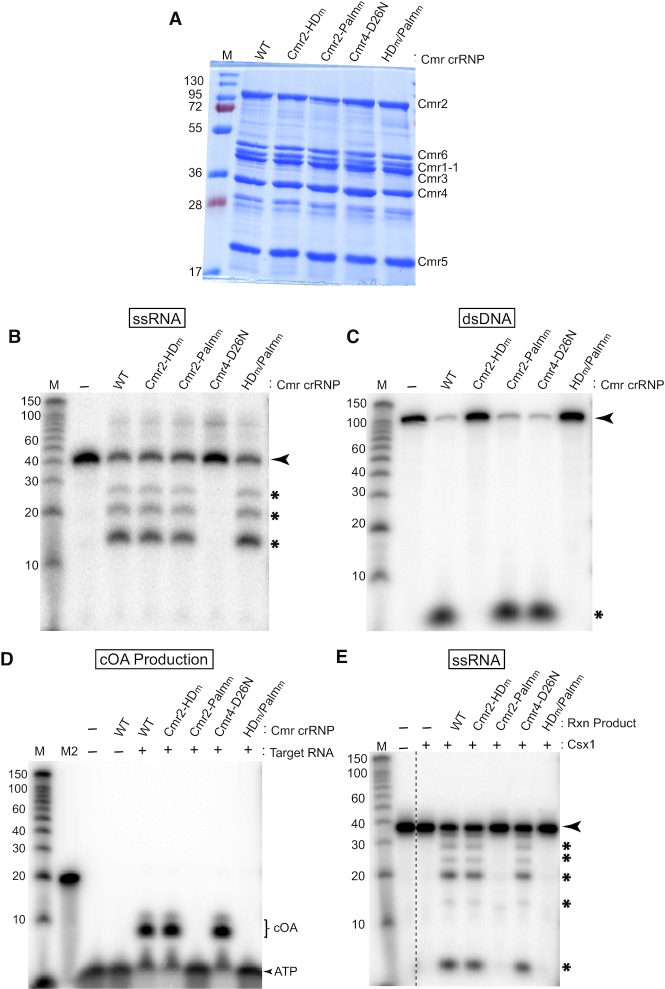
The ribonuclease activity of *Pfu* Csx1 is activated by cOA species produced by the *Pfu* effector complex in a Cmr2 Palm domain-dependent manner. (**A**) Purification of *Pfu* complex proteins. HD and Palm mutations are located within Cmr2. The D26N mutation is located within Cmr4. (**B**) RNase activity of each *Pfu* Cmr complex. Radiolabeled 7.01 Target RNA was incubated with Cmr complexes and reaction products were visualized by urea-PAGE. Radiolabeled RNA size standards (M) in nucleotides were used. The black arrow indicates the full-length substrate and the black asterisks indicate cleavage products. (**C**) DNase activity of each *Pfu* Cmr complex. Each complex was incubated with dsDNA (label located on DNA Target 2) and reactions were visualized as described in part B. (**D**) cOA production activity of the *Pfu* Cmr crRNP. The five Cmr complexes were tested for cOA production in presence (+) and absence (–) of 7.01 Target RNA. Reaction products were run on a denaturing gel. A radiolabeled alkaline hydrolysis ladder (M2) and size standard (M) were added to the urea-PAGE analysis. (**E**) Activation of wildtype Csx1 by *Pfu* cOAs. Csx1 was tested for ribonuclease activity under activating (20 nM Csx1) conditions. Reactions were visualized as described in part B.

Next, we addressed whether the *Pfu* Cmr system functioned through generating cOA signaling molecules as has been observed for other bacterial and archaeal Type III systems (31–33). *In vitro* reconstituted Cmr crRNPs were assayed for their ability to generate cOA compounds as well as to support previously observed RNase and DNase activities (Figure 2) (9,17). Wildtype as well as four different functional mutants of the Cmr crRNP complex (Cmr2-HD_m_, Cmr2-Palm_m_, Cmr4-D26N and Cmr2-HD_m_/Palm_m_), were assembled *in vitro* (Figure 2A) and tested. As expected, target RNAse activity was observed for all Cmr crRNP complexes except those harboring a mutation in the Cmr4 subunit (Cmr4-D26N) (Figure 2B). Moreover, DNase activity was only observed for wildtype, Cmr2-Palm_m_, and Cmr4-D26N complexes but not Cmr2 mutants in which the HD motif was mutated (Figure 2C). To test for cOA production, the same five complexes were incubated with α-^32^P-ATP and the products of the reactions were separated by denaturing polyacrylamide gel electrophoresis. Conversion of the α-^32^P-ATP to slower migrating products indicative of cOA compounds was only observed for complexes with an intact Cmr2 Palm GGDD motif. Additionally, the presence of the target RNA was required for cOA production (Figure 2D). These findings reveal that the *Pfu* Type III-B Cmr system possesses the highly conserved activity of cOA generation which is catalyzed by the Cmr2 Palm GGDD motif and is dependent upon interactions between crRNPs and complementary target RNA.

### *Pfu* Csx1 ribonuclease activity is activated by cyclic oligoadenylate produced by the Cmr complex

Next we sought to determine if the cyclic oligoadenylate compounds produced by *Pfu* Cmr crRNPs, are capable of stimulating the ribonuclease activity of *Pfu* Csx1 *in vitro* (Figure 2E). Previously, we found that recombinant *Pfu* Csx1 is capable of cleaving RNA substrates via the HEPN motif but only when high concentrations (e.g. 500 nM) of the Csx1 enzyme is used (37). Therefore, we used levels of the Csx1 protein (20 nM) that showed no or low RNase capacity to test if cOA produced by Cmr crRNPs could stimulate the RNase activity of Csx1. ATP (unlabeled) was incubated with wildtype and mutant Cmr crRNPs and the products of the reactions were added to reactions containing Csx1 and radiolabeled substrate RNA (Figure 2E). Cleavage of the radiolabeled substrate RNA by Csx1 depended upon Cmr crRNP complexes having an intact Cmr2-Palm GGDD motif (Figure 2D and E). The results indicate that Cmr2 Palm domain-mediated cOA production by the Cmr crRNP complex is a potent activator of the RNA degradation capacity of *Pfu* Csx1.

### *Pfu* Csx1 is activated by cA_4_ species produced by Cmr complexes

**Figure 3.**
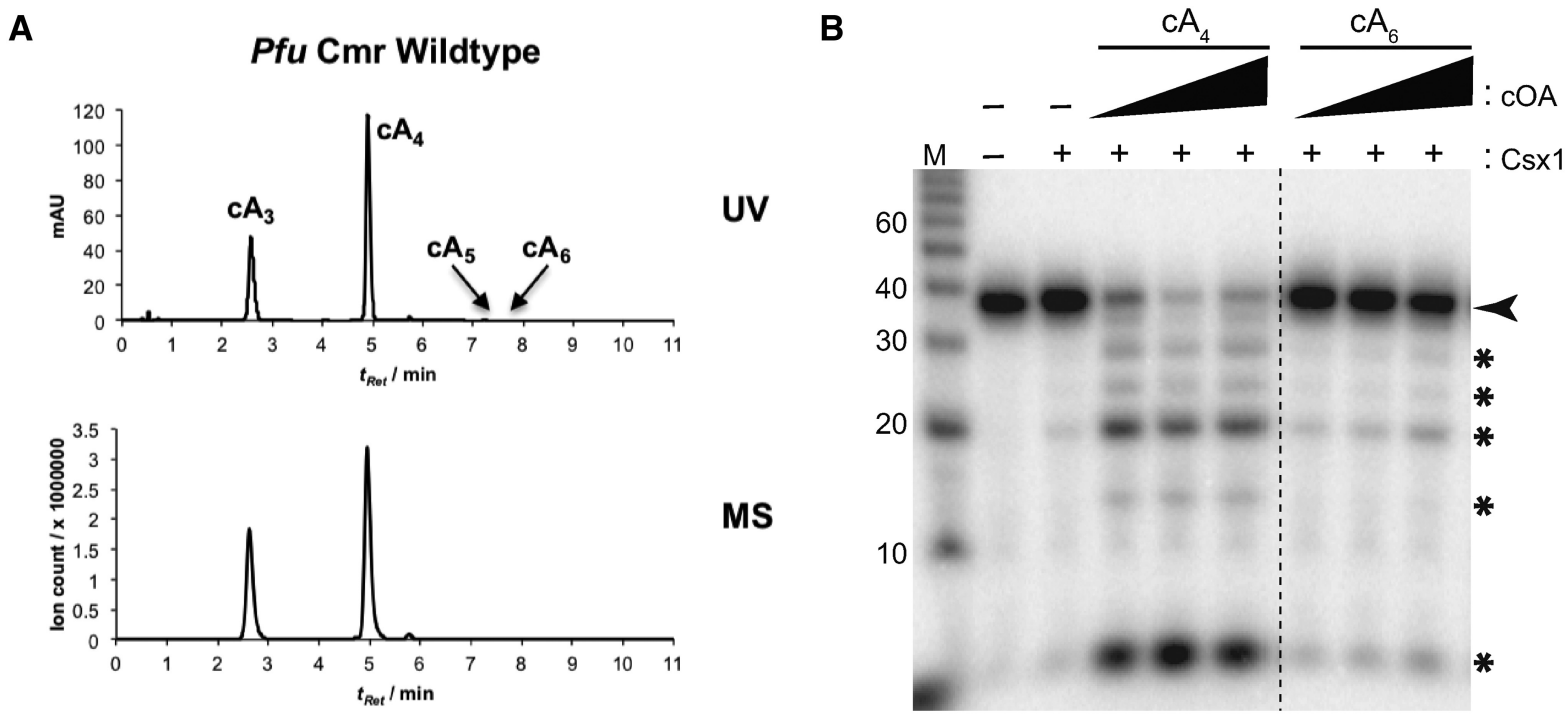
cA_4_ is the relevant activator for *Pfu* Csx1. (**A**) UV chromatogram (258 nm) and MS chromatogram (extracted ion chromatogram for *m/z* 494.6, 659.1, 823.6, 988.2) for cA samples from *Pfu* Cmr wildtype complexes. (**B**) *Pfu* Csx1 activation assays with synthetic cOA. Wildtype Csx1 (20 nM) was incubated with increasing concentrations of synthetic cA_4_ and cA_6_. For each activator, 1.85, 18.5 and 185 pM concentrations were used. A radiolabeled alkaline RNA size marker (M) was added to the urea-PAGE analysis. The black arrow indicates the full-length RNA substrate and the black asterisks indicate cleavage products. Dotted lines are indicative of noncontiguous data being omitted from the gel.

The identity of the compounds generated after addition of ATP to wildtype Cmr complexes (Figure 2D) was determined by mass spectrometry using established methods (41). We found that the *Pfu* Cmr complex primarily produces cyclic-triadenylate (cA_3_) and cyclic-tetraadenylate (cA_4_) species (Figure 3A). The data reveal that cA_4_ is the most abundant species and is approximately twice as abundant as cA_3_; there were traces of cA_5_ and cA_6_ which made up less than 5% of the total cOA species. To address which form(s) of the generated cOA leads to the activation of Csx1 ribonuclease activity, varying concentrations of commercially available synthetic cA_4_ and cA_6_ compounds were incubated with *Pfu* Csx1 in the presence of 5′-end labeled substrate RNA (cA_3_ and cA_5_ compounds were not commercially available and so could not be tested). cA_4_ but not cA_6_ stimulated the ribonuclease activity of Csx1 (Figure 3). These results indicate that cA_4_ is both the dominant cA species produced by *Pfu* Cmr effector complexes and a potent activator of *Pfu* Csx1 RNA cleavage activity.

### The CARF domain of *Pfu* Csx1 is needed for cOA ribonuclease activation

**Figure 4.**
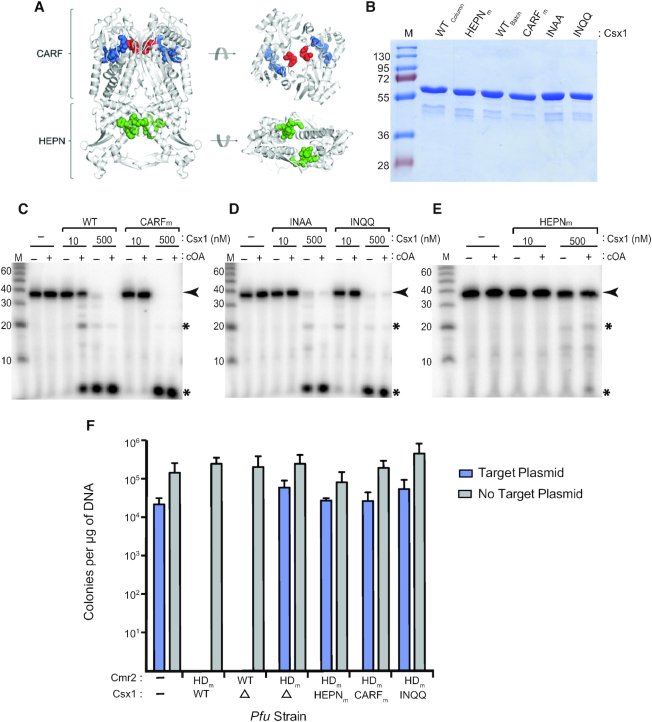
Activation of *Pfu* Csx1 is CARF domain dependent. (**A**) Ribbon structure of *Pfu* Csx1 (PDB: 4EOG) indicating the CARF and HEPN domains. Residue H436 was mutated in the HEPN domain (green). Residues 121–127 were deleted from the CARF domain (blue), and residues I169 and N170 were mutated within the CARF domain (red). (**B**) SDS-PAGE analysis of *Pfu* Csx1 protein purifications. WT_column_ refers to protein purified using a column method and WT_batch_ refers to protein purified using a batch method. (**C–E**) *In vitro* activation of Csx1 mutants. Wildtype Csx1 along with four Csx1 mutants (CARF_m_, INAA, INQQ, HEPN_m_) was tested for ribonuclease activity under high (500 nM Csx1) and low (10 nM Csx1) concentration conditions. A radiolabeled alkaline RNA size marker (M) was added to the urea-PAGE analysis. The black arrow indicates the full length RNA substrate and the black asterisks indicate cleavage products. (**F**) *In vivo* plasmid silencing assay results for CARF domain mutants. Strains were transformed with the target plasmid (blue) or no target plasmid (gray). Mean colonies obtained per μg of plasmid DNA are plotted for two replicates. Error bars indicate standard deviation of replicates.

The X-ray structure of *Pfu* Csx1 has been solved (63) and reveals two major domains that are conserved amongst Type III-affiliated Csx1 or Csm6 RNases: the HEPN (RNase active site) and CARF domains (shown in other systems to selectively bind cOA for allosteric activation of the HEPN RNase activity) (Figure 4A). Previously, we found that mutation to the HEPN motif (Csx1-HEPN_m_; H436A) disrupted the ribonuclease activity of *Pfu* Csx1 (37). To investigate if the CARF domain is required for the cOA-stimulated RNase activity of *Pfu* Csx1, we examined the effects of Csx1 mutants predicted to disrupt CARF function and cOA binding (Figure 4A and B). Csx1-CARF_m_ contains a deletion of residues 121–127 predicted to be critical for CARF domain assembly. The Csx1-INAA and Csx1-INQQ mutants are predicted to prevent specific binding of cOA and contain a double mutation of residues Isoleucine169 and Asparagine170 to alanine or glutamine (Figure 4A and Supplemental Figure S3). We previously showed that high (500 nM) concentrations of wildtype Csx1 cleave RNA without cOA activation (37). We further analyzed *Pfu* Csx1 activity and determined that lowering the concentration to 10–20 nM resulted in a loss of detectable RNase activity by Csx1 in the absence of cOA (Figure 4). All three Csx1 CARF mutants disrupted the ability of low levels of Csx1 (10 nM) to support RNA cleavage activity in response to cOA (Figure 4C and D) as is observed for the wildtype Csx1 enzyme (Figure 4C). None of the CARF mutants impaired the ability of Csx1 to cleave RNAs when high amounts of enzyme (500 nM) were assayed showing that the mutations *per se* did not negatively impact HEPN functionality (Figure 4C and D). In contrast, the Csx1-HEPN_m_ mutant failed to efficiently cleave RNA at either low (10 nM) or high (500 nM) concentrations and with or without cOA addition (Figure 4E).

The impact of CARF domain mutations was further tested *in vivo* on anti-plasmid immunity (Figure 4F). We observed that, when paired with a Cmr2-HD_m_ mutation that disrupts DNA cleavage, the Csx1-CARF_m_ and Csx1-INQQ mutants both led to a loss of anti-plasmid immunity (Figure 4F). As expected, wild type Csx1 but not the Csx1-HEPN_m_ supported anti-plasmid immunity. Collectively, the *in vitro* and *in vivo* results support a key role for the CARF domain of *Pfu* Csx1 in triggering cOA-stimulated RNase activity and reveal important CARF domain residues responsible for mediating cOA-triggered, *Pfu* Csx1 RNase activation.

### *Pfu* Csx1 cleaves and inactivates cOA using its adenosine-specific HEPN RNase active site

**Figure 5.**
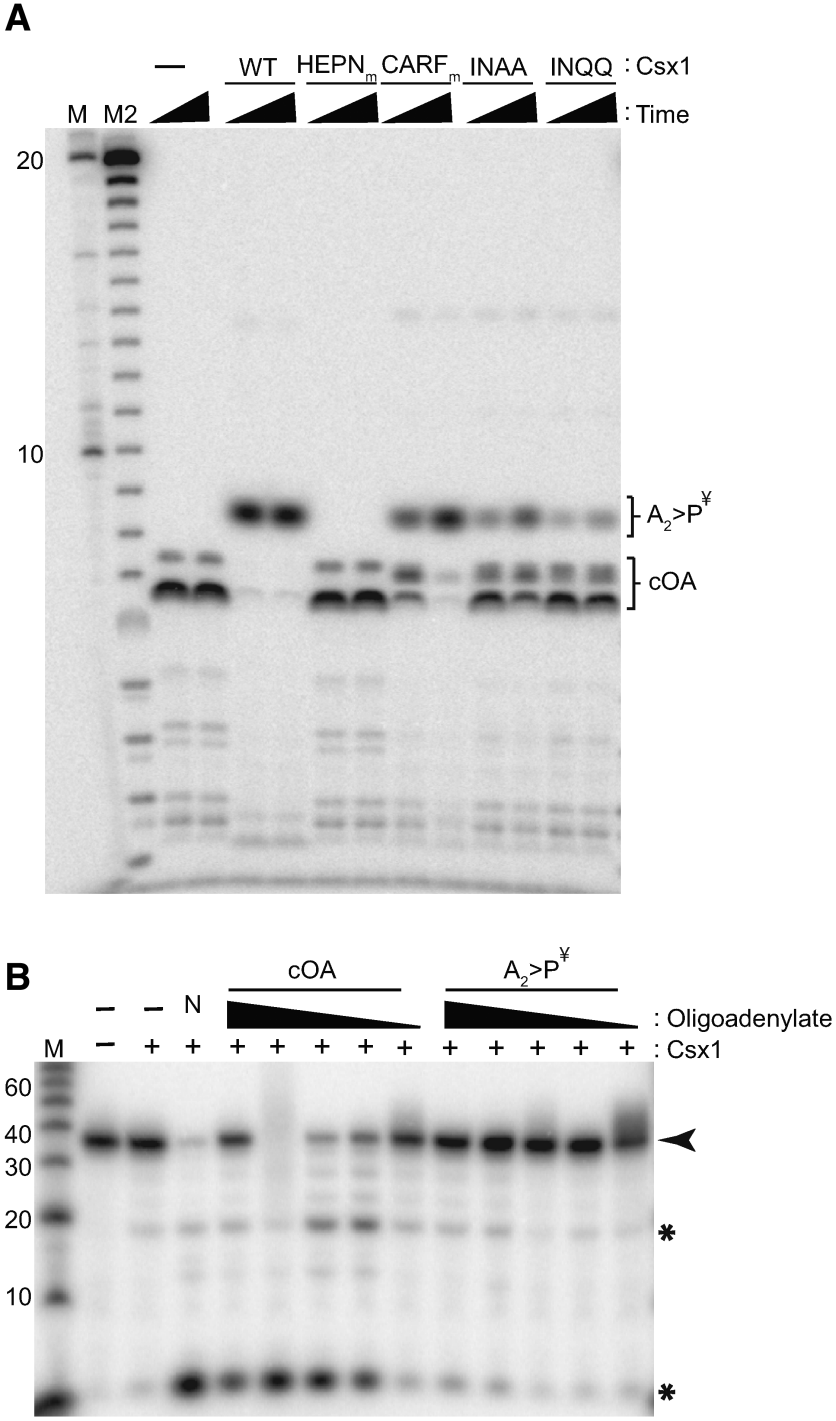
Degradation of cOAs by *Pfu* Csx1 inactivates the cOA activators. (**A**) Sequencing gel reactions of Csx1 proteins incubated with *Pfu* cOAs. Five different *Pfu* Csx1 proteins were incubated with radiolabeled cOAs for two timepoints-5 and 20 mins. Urea-page analysis on sequencing gels was performed. Radiolabeled RNA size marker (M) and alkaline hydrolysis ladder (M) and were included in the analysis. (**B**) Effect of Csx1 treatment on cOA activation activity. Unlabeled *Pfu* cOAs were treated with 600 nM of wildtype Csx1 or no protein for 30 min. The plus extraction set of reactions were tested for activation of wildtype Csx1 by incubating 20 nM of wildtype Csx1 with decreasing concentrations of A_2_>P^¥^ or cOA_4_— roughly 1.25, 0.6, 0.6 × 10^−1^, 0.6 × 10^−2^, and 0.6 × 10^−4^ uM. ¥ indicates a putative designation for this reaction product. Native cOA without the 30 min incubation or extraction was utilized as a control (N).

Once cOAs are produced by Type III crRNPs, it is not well understood how cOA levels are controlled to prevent unnecessary destruction of vital cellular RNAs by trans-acting Csx1 (or related Csm6) that could lead to host cell toxicity or death during the immune response. Recently, a new class of CARF-domain containing proteins called ring nucleases, were discovered in *Sulfolobus solfataricus* and found to exhibit cOA nuclease activity that halts cOA-triggered Csx1 activity (45). *S. solfataricus* Csx1 itself was not able to degrade cOA molecules (45). In contrast, Csm6 of *Thermococcus onnurineus* exhibited an intrinsic ability to utilize its CARF domain to both bind and cleave cOA_4_ which generates inactive, linear di-adenylate products with 2′,3′ cyclic phosphate termini (A_2_>p) (44). Previous characterization of *Pfu* Csx1 revealed an adenosine specificity for endoribonuclease activity conferred by the HEPN ribonuclease motif (37). That knowledge combined with the lack of a CARF domain-containing ring nuclease homologs in *P. furiosus*, led us determine if *Pfu* Csx1 was capable of controlling its own ribonuclease activity by recognizing and degrading cOA (Figure 5). Indeed, we found that wildtype Csx1 protein efficiently converted native cOA substrates into products with relative mobilities consistent with inactive linear di-adenylate (A_2_ > p) products (Figure 5A and (43,45)). The conversion of cOA into the presumed A_2_>p products was abolished by mutations in the HEPN domain but not mutations in the CARF domain (Figure 5A). Moreover, we found that the Csx1-mediated cOA cleavage products failed to activate Csx1 ribonuclease activity (Figure 5B). The results indicate the adenosine-specific *Pfu* Csx1 endoribonuclease responsible for target RNA destruction (37), also utilizes its HEPN active site to recognize, cleave and inactivate cOA molecules providing an autoregulation negative feedback control mechanism that limits Csx1 RNase activity.

### Activation of Cmr2-mediated DNA nuclease activity and cOA production is dependent upon distinct target PFS elements

**Figure 6.**
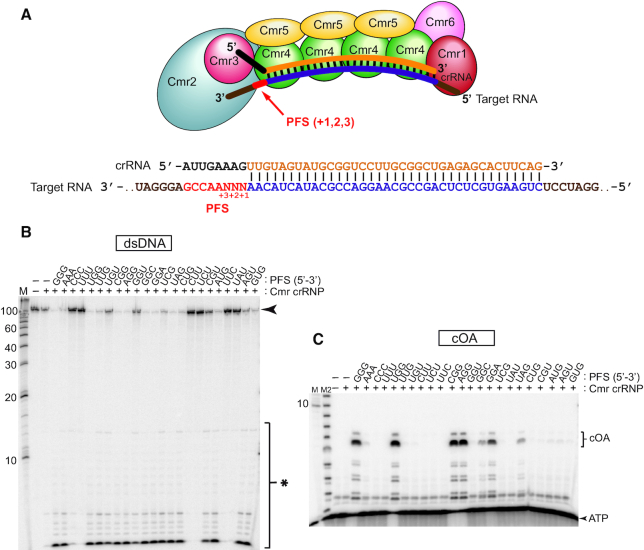
*Pfu* Cmr DNase and cOA production activities are PFS dependent. (**A**) Composition of the *Pfu* Cmr crRNP. The Cmr effector complex is composed of Cmr1–6 and a mature crRNA (black & orange) containing a 5′ tag (black). The *Pfu* Cmr complex is activated by binding of complementary target RNA (purple) to the crRNA. The target RNA contains a PFS (red) within it that is located adjacent (3′) to the target sequence. (**B**) Target RNA dependent activation of DNase activity by the *Pfu* complex. Cmr crRNP complexes were incubated with radiolabeled double-stranded DNA in the presence of 23 different target RNAs. Each target RNA contains a different three nucleotide sequence in the PFS region of the target RNA. Urea-page analysis on sequencing gels was performed. Radiolabeled RNA size markers (M) were included in the analysis. The black arrow indicates the full-length RNA substrate and the black asterisks indicate cleavage products. (**C**) Target RNA dependent activation of cOA production activity by the *Pfu* complex using the same target RNAs mentioned in part B. Products were analyzed as described in part B. An alkaline hydrolysis ladder (M2) was included in the urea-PAGE analysis.

Prior investigation into regulatory mechanisms controlling the function of *Pfu* Cmr complexes revealed the important role of three nucleotides within the PFS immediately adjacent to the crRNA/target RNA protospacer interaction (see Figure 6A) (9). Successful anti-plasmid immunity *in vivo* and Cmr2-mediated DNA cleavage (via the HD domain) *in vitro* required a PFS with a NGN, NNG, or NAA sequence (9) and Table 1. In contrast, target RNA cleavage by *Pfu* crRNPs (via Cmr4 backbone subunit) occurs independent of the PFS (9,11). Given the newly observed cOA generation activity for *Pfu* Cmr complexes revealed in this study (Figure 2D), we examined whether the first three positions of the PFS were important for cOA production. Specifically, we addressed whether a large panel of target RNAs that differed only in having distinct PFS elements could activate DNA cleavage (Figure 6B) or cOA production (Figure 6C) *in vitro* in parallel reactions. As expected, only target RNAs containing a NGN, NNG, or NAA protospacer flanking sequence activated DNA cleavage (Figure 6B). In contrast, a much smaller subset of the same target RNAs activated cOA production (Figure 6C). The target RNAs eliciting strong cOA production activity contain a PFS consensus of NGR sequence. Weak cOA generation activity was observed for target RNAs containing a GGC or UAG sequence within the first three positions of the PFS. A summary of the results for all PFS elements tested on either DNase or cOA generation activities are provided in Table 1. The results reveal a difference in specificity for PFS elements needed for activating cOA generation vs. DNase activity for the *Pfu* Cmr crRNP.

**Table 1.** Summary of PFS requirements for *Pfu* Cmr activities

Target RNA PFS (5′-3′)		CRISPR defense *(in vivo)*	DNase *(in vitro)*	cOA *(in vitro)*
**NNN**	GGG	+	+	+
	AAA	+	+	**−**
	CCC	**−**	**−**	**−**
	UUU	**−**	**−**	**−**
**NGG**	GGG	+	+	+
	AGG	+	+	+
	CGG	+	+	+
	UGG	+	+	+
**GGN**	GGG	+	+	+
	GGA	+	+	+
	GGC	+	+	(+)
	GGU	+	+	**−**
**UNG**	UGG	+	+	+
	UAG	+	+	(+)
	UCG	+	+	**−**
	UUG	+	+	**−**
**NUG**	GUG	+	+	**−**
	AUG	+	+	**−**
	CUG	+	+	**−**
	UUG	+	+	**−**
**NGU**	GGU	+	+	**−**
	AGU	+	+	**−**
	CGU	+	+	**−**
	UGU	+	+	**−**
**UNU**	UGU	+	+	**−**
	UAU	**−**	**−**	**−**
	UCU	**−**	**−**	**−**
	UUU	**−**	**−**	**−**
**YUY**	CUU	**−**	**−**	**−**
	UUC	**−**	**−**	**−**

Recap of PFS dependent CRISPR defense, DNase activity, and cOA production activity results observed for target RNAs used in this study and previously reported (9). + indicates the activity was observed, – denotes the activity was not observed, and (+) indicates the activity was weakly observed.

## DISCUSSION

**Figure 7.**
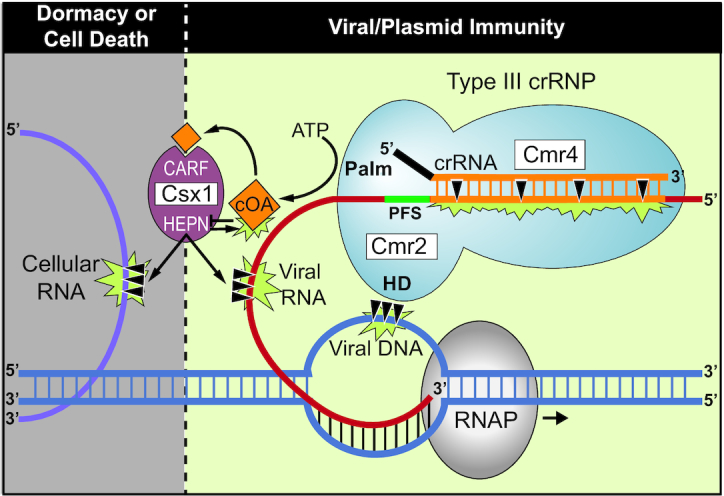
Model for regulation of the RNAse, DNase, and cOA synthetase activities of the *Pfu* Type III-B effector crRNP in providing anti-viral immunity. Inactive Type III-B crRNPs become activated upon interaction with expressed viral RNA. crRNA-guided base-pairing to the viral RNA and Cas protein recognition of the PFS results in conformational changes that trigger three important activities: viral RNA cleavage by Cmr4, viral DNA cleavage by the HD domain of Cmr2 (a member of the Cas10 superfamily), and ATP dependent production of cyclic oligoadenylates (cOA) by the Palm domain of Cmr2. The cOA second messengers allosterically regulate the trans-acting Csx1 enzyme by binding to the CARF domain to trigger HEPN RNase activity. Clearance of the viral DNAs and RNAs provides immunity and restores the crRNPs back to the inactive state. Under conditions of controlled immunity, Csx1 degrades and inactivates cOA signaling molecules using the HEPN domain, to provide an auto-inhibitory off-switch of Csx1 RNase activation. Inefficient degradation of cOA molecules by the Csx1 or failure to efficiently destroy the viral RNAs or viral genome may cause Csx1 to destroy both viral and cellular RNA molecules, leading to cellular dormancy or death.

The *Pyrococcus furious* Type III-B (Cmr) effector crRNP was the first example of a CRISPR system that identifies and pairs with RNA transcripts rather than DNA strands of invaders (17). A decade of subsequent *in vivo* and *in vitro* research has revealed the detailed structure and organization of the *Pfu* Cmr effector crRNPs (20,21,30,64,65) and determined that the system employs highly versatile strategies to combat invading mobile genetic elements (Figure 7). Previous work showed that immunity provided by the *Pfu* Cmr crRNP effector complexes, requires that the invasive DNA undergo transcription to produce the target RNA required for triggering destruction of both the target RNA transcript and invading genome by intrinsic Cmr crRNP RNase (Cmr4) and DNase (Cmr2 HD domain) activities, respectively (9,11,17,27). Here we demonstrate that *Pfu* Cmr effector crRNP complexes also produce cyclic oligoadenylate second messenger compounds that amplify the immune response by activating the trans-acting Csx1 ribonuclease via their CARF domains (Figures 2-4). Furthermore, the Csx1 HEPN RNase active site, responsible for target RNA destruction via cleaving after adenosine residues (37), also degrades its cognate cyclic-tetra-AMP (cA_4_) activator to switch off the signaling pathway and to limit the activity of Csx1 through autoregulation (Figures 5 and 7). Our *in vivo* mutational analyses show that either the DNase activity (via HD domain of Cmr2) or RNase activity (via HEPN motif of Csx1 and triggered by cOA generation by the GGDD motif of Cmr2 Palm domain using ATP precursors) leads to robust anti-plasmid immunity. Our finding that the DNase and cOA generation capacities are activated by distinct 3′ protospacer flanking sequences (PFS) of the target RNA highlight that the two target-RNA stimulated enzymatic processes are differentially regulated by distinct allosteric control mechanisms. Taken together with results obtained from other investigated bacterial or archaeal Type III-B (Cmr) and III-A (Csm) systems, our results contribute to making a compelling case for Type III systems as the most complex and highly regulated CRISPR systems discovered thus far.

### Role of trans-acting Csx1 endoribonuclease in conferring anti-plasmid immunity

Our previous investigation into the requirement of *Pfu* Csx1 in anti-plasmid immunity through single *csx1* gene mutational analysis, failed to uncover its role (9). The more comprehensive gene mutational analysis performed here reveals that the Csx1 RNase activity or the Cmr2 DNase activity each can independently provide robust anti-plasmid immunity (Figure 1). We show that immunity remains unchanged by disrupting either the DNase activity of the *Pfu* crRNP (via mutation of the HD motif of Cmr2), the RNase activity of Csx1 (via mutation of the HEPN motif or csx1 gene deletion), or the ability to generate cOA second messengers (via the Palm domain of Cmr2) needed to activate Csx1 RNase activity (Figure 1). In contrast, double mutations that block both DNase and Csx1 RNase activity (either directly or through interfering with cOA signaling), are required to prevent immunity (Figures 1 and 4). The results reveal that Cmr2 DNase and Csx1 RNase activities serve a redundant function in conferring highly effective anti-plasmid immunity by the Type III-B *Pfu* Cmr system. In comparison, other studied Type III-A or III-B systems have shown a dominant role for Csm6/Csx1 activity (32,34,41,47,62) or Cmr2/Csm1 DNase activity (14,35,38,50,62) to execute effective immunity against plasmids (14,34,38,47,62) or phages (32,35). Type III-C, D, E and F systems are predicted to contain Cmr2 or Csm1 (i.e. Cas10) subunits that lack functional DNase or cOA generating capacities and further work on these systems is required to understand if and how they execute immunity against mobile genetic elements (3,4).

The exact mechanism of action of *Pfu* Csx1 for anti-plasmid immunity remains to be determined. For example, it is unclear if *Pfu* Csx1 functions through selectively destroying the invading target RNA (to provide specific immunity) and/or by destroying host RNA transcripts (leading to cellular dormancy or death) (Figure 7). We are unable to distinguish these two different scenarios given that inducible gene expression systems have yet to be established for *Pfu* that would enable testing which RNAs are being destroyed as a function of activation of the Cmr crRNP activities over time. Given that *Pfu* Csx1 was shown to cleave various RNAs without specificity *in vitro* (except that it cleaves after adenosine residues (37)) and that Csx1 or related Csm6 are not normally stably associated with Type III effector crRNPs (15,17,21–23,34), to be selective for the target RNA would involve conditional recruitment of Csx1 to the crRNP when the complex is engaged in interaction with the target RNA protospacer. The related Csm6 RNase from the Type III-B system of *S. epidermidis* was found to either selectively cleave target RNA (including at regions outside of the RNA protospacer segment) while sparing cellular RNAs (35), or to destroy both target RNA and cellular RNAs (38). It is conceivable that low levels of target RNA and second messenger signaling may limit Csx1 and Csm6 RNase activity to target RNA destruction, while elevated target RNA concentrations and high cOA signaling may lead to more indiscriminate RNA degradation. Selective targeting of invader RNAs would promote immunity and maintain viable host cells. In contrast, indiscriminate RNA destruction would prevent the growth or kill infected host cells, which would effectively prevent the spread of the invader to other host cells in the local environment (Figure 7).

### Second messenger signaling pathway leading to Csx1 RNase activation and deactivation

We show for the first time that the *Pfu* Cmr complex generates primarily cA_4_ signaling molecules (Figure 3A) that significantly stimulate the RNase activity of Csx1 (Figures 2-4). Either cA_4_ or cA_6_ have been found to be the relevant activators for various characterized bacterial or archaeal Csx1 and Csm6 enzymes (31–33,41,42,44,51). Interestingly, the HEPN RNase active site of *Pfu* Csx1 (which is adenosine-specific) cleaves and inactivates cOA signaling molecules (Figure 5) in addition to executing the destruction of adenosine-containing, target RNA transcripts (Figures 2–5 and (37)). Previous studies have shown that the CARF domain is the cOA sensor domain (31,32,44,46,51) and accordingly, mutation of the CARF domain of *Pfu* Csx1 blocks activation of Csx1 RNase activity. However, *Pfu* Csx1 CARF domain inactivation did not abolish cleavage and inactivation of cOA second messengers (Figure 5).

Our finding that the HEPN RNase motif, rather than CARF domain of *Pfu* Csx1, can cleave and inactivate cOA signaling molecules expands the number of mechanisms capable of down-regulating Type III-induced cOA signaling pathways. For example, *Sulfolobus islandicus* Csx1 appears to lack the ability to itself degrade cOA_4_ molecules and instead utilizes extrinsic CARF domain-containing factors (called ring nucleases) to switch off Csx1 RNase activation (45). Similar to our finding with *Pfu* Csx1, Csm6 from another hyperthermophlic archaeon, *Thermococcus onnurineus (Ton)*, was found to be capable of autoregulating RNase activity through binding and cleaving cA_4_ (44). However, unlike *Pfu* Csx1, *Ton* cA_4_ cleavage of cA_4_ into inactive linearized adenosine dinucleotide products (A_2_>P) depended upon the CARF rather than HEPN domain despite evidence from high resolution structural studies showing that cOA_4_ binds to both CARF and HEPN domains of *Ton* Csm6 (44). Interestingly, the HEPN domains of *Ton* Csm6 and *Pfu* Csx1 each cleave RNA with a strict adenosine specificity (37,44). Furthermore, *Thermus thermophilus* Csm6 exhibits an intrinsic ability to degrade its cognate cA_4_ using its CARF domain but not its HEPN domain (43).

Studies examining RNA cleavage patterns of various Csx1 and Csm6 HEPN endoribonucleases revealed that many are either specific for cleaving after adenosines (37,44) or purines (adenosines and guanosines) (31,34). These catalytic properties indicate that other Csx1 or Csm6 enzymes may utilize their HEPN RNase active site to degrade cOA molecules. Thus, *Pfu* Csx1 HEPN domain-mediated destruction of its cognate cOA activator reveals a novel and distinct auto-catalytic control mechanism that limits Csx1 RNase activity that may be common to other Type III systems.

### Influence of target RNA interactions in controlling DNase and RNase activities of *Pfu* effector crRNPs

All characterized Type III CRISPR–Cas systems are specifically activated through identifying and interacting with target RNA. Base-pairing interactions between crRNAs within crRNP effector complexes and matching target RNA protospacer elements, has been shown to trigger major conformational changes within Type III complexes (25,49,50). In turn, less understood, minor target RNA-induced conformational changes lead to activation of several enzymatic activities of the complexes including RNase, DNase and cOA generation needed for downstream activation of Csx1 and Csm6 HEPN endoribonucleases (25,49,50,52).

Some but not all of these enzymatic activities are further regulated by a ∼eight base-pair PFS region of the target RNA that is located just 3′ of the target RNA protospacer region. For all Type III systems evaluated thus far, including *Pfu* (9), if the target RNA PFS is complementary to the 5′ tag of the crRNA (as is the case if anti-sense transcription of the CRISPR locus occurs (27,66)) then base-paring between residues of the crRNA tag and the PFS of the target RNA apparently prevents the ability of the crRNPs to allosterically activate DNase and cOA production (9,10,12–14,54,67,68). In contrast, cleavage of the target RNA (at regular 6 nucleotide intervals throughout target RNA protospacer region) by Cmr4 (III-B) or Csm4 (III-A) crRNP backbone RNases is unaffected by crRNA 5′ tag/target RNA PFS interactions (9,11) (Figure 7).

There are apparent differences in PFS requirements for activation of DNase and cOA generation activities for distinct Type III systems. For example, a simple lack of base-pairing between 5′ crRNA tag and target RNA PFS, appears to be sufficient for activating these enzymatic activities for the *Staphylococcus epidermidis* Type III-A system (69). In contrast, the identity of PFS sequences, especially within the +1, +2 and/or +3 positions have been found to be important for conferring immunity for Type III systems of *Pyrococcus furiosus* (9), *Streptococcus thermophilus* (25,48) and *Thermotoga maritima* (54). For most studied Type III systems, the PFS requirements have not been thoroughly investigated.

Based on recent Type III crRNP structural studies, the PFS sequences in the +1, +2, +3 positions are predicted to interact with Csm1/Cmr2 (Cas10) and Csm4/Cmr3 (5′ tag interacting) subunits (25,48,49). We propose that in systems where the identity of protospacer flanking sequences matter for activating function such as *Pfu*, PFS RNA/protein contacts play a key role in initiating the conformational changes that ultimately trigger activation of the Cas10 HD domains to cleave invader DNA and the Cas10 Palm domain GGGDD motif to convert ATP into cOA second messengers. Interestingly, our systematic analyses of the impact of varying the PFS elements in the +1, +2 and +3 positions of the target RNA, revealed that a given PFS can activate DNase activity, cOA production, both activities or neither activity (independent of the ability of these PFS nucleotides to base-pair with the 5′ crRNA tag element) (Figure 6 and Table 1). Furthermore, we found a direct correlation between PFS elements that were previously found to support anti-plasmid immunity *in vivo* (9) and those found to also be required for in DNase activity *in vitro* (Figure 6 and Table 1). In contrast, only a subset of PFS elements required *in vivo*, resulted in cOA production *in vitro* (Figure 6 and Table 1).

All *Pfu* PFS elements that triggered activity, shared a purine-rich character within position +2 and +3 of the target RNA PFS appears to responsible for differentially activating the DNase or cOA synthesis. These results have provided insight that the two important activities for immunity are differentially controlled by distinct molecular mechanisms. Additional evidence supporting the notion that distinct conformational changes triggered by PFS/protein interactions separately influence either DNase or cOA production were observed with *S. thermophilus* Type III-A system where Csm1 mutations at residues thought to transmit allosteric effects of the PFS were found to impair DNase activity only, cOA synthesis only, or both activities (25). Of note, the *Pfu* spacer acquisition machinery responsible for addition of new spacers into CRISPR arrays, recognized the invading DNAs having a 5′-NGG-3′ consensus PAM (protospacer adjacent motif) (70,71). In turn, our results predict that the resultant 5′-NGG-3′ RNA PFS of expressed invader genomes would trigger both DNA cleavage and cOA activities of *Pfu* Cmr effector crRNPs (Figure 6 and Table 1). A functional coupling between PAM recognition during CRISPR spacer acquisition and Type III interference activation may boost the specificity of certain Type III systems to compensate for an observed high degree of tolerance to mismatches in the rest of the target RNA (69).

### Role for the RNAse activity of Cmr4 subunit?

We were unable to determine the possible *in vivo* role of the RNase activity of Cmr4 on anti-plasmid immunity. Cmr4 is the backbone subunit of the III-B crRNP (see Figure 1) that cleaves the bound target RNA at regular six nucleotide intervals (27). Despite repeated attempts, we failed to generate a strain in which the RNase activity of Cmr4 was inactivated (via D26N mutation that blocks target RNA cleavage *in vitro* (Figure 2)). Given that we were able to readily create the catalytically inactive Cmr4 mutant in a strain in which both the DNase and Csx1 RNase activities were also inactivated (i.e. the Cmr2-HD_m_/Csx1_Δ_ strain that cannot execute anti-plasmid immunity (Figure 1)), we suspect that preventing Cmr4 RNase activity results in host cell lethality brought about host cell DNAase activity and/or cOA signaling and Csx1 RNase activation as the result of generation of constitutively active Type III-B crRNPs. This would occur if there was recognition of endogenous P. furiousus RNA(s) exhibiting complementarity to any of the 200 known crRNAs that are assembled into *P. furious* type III-B crRNPs (55,56). Consistent with this possibility, it has been shown that cleavage of the crRNA-bound target RNA by Cmr4 (and related Csm3 protein of III-A crRNPs) stimulates rapid release of the target RNA into solution to temporally control (switch off) the DNase and cOA production activities of the complex (9,10,13). Failure to cleave the target RNA that is required for triggering the activities of the complex, is expected to drive the effector crRNPs into a long-lasting nuclease-active state. The scenario is further supported by findings with the *S. epidermidis* III-A complex that exhibits a hyperactive immunity phenotype *in vivo* when target RNA cleavage was disrupted through inducible expression of the comparable catalytically inactive Csm3 mutant (14). Taken together, we view it likely that the RNase activity of *P. furiousus* Cmr4 normally contributes to both the destruction and turnover of the target RNA and plays an important role in immunity and regulation of type III-B crRNP activity.

### Contribution of DNase vs. RNase activities to type III immunity

The ability of type III CRISPR–Cas systems to act both at the level of DNase and RNase degradation for invader immunity, sets them apart from all know other CRISPR types. Moreover, it is clear that there is great variation in the relative importance of these two distinct nuclease activities in conferring immunity between different type III systems as well as within a system for targeting specific invaders. Here, we find that anti-plasmid immunity for the *P. furiosus* III-B system is robust when only employing DNase activity (mediated by the HD domain of Cmr2) or RNAse activity (mediated by the cOA-regulated, HEPN domain of Csx1) (Figure 1). This is in contrast to *in vivo* results obtained for several other type III systems including *Sulfolobus islandicus* (47), *S. epidermidis* (62,72), *S. thermophilus* (72), *Lactococcus lactis* (72) shown to rely primarily on Csx1 RNase activity given that plasmid immunity was abolished by single deletion or mutational inactivation of the csx1 or csm6 gene. Yet other systems appear to solely utilize the DNase activity as exemplified by the recently characterized *Lactobacillus delbrueckii* found to confer anti-plasmid immunity despite lacking a Csm6 gene and not being capable of producing cOA (73). Anti-plasmid immunity for the *S. aureus* III-B system, like *P. furious*, appears to depend on both DNase and RNase targeting but a partial interference phenotype is observed with csx1 gene knockout, indicating relatively weak DNase activity (74). Finally, when anti-viral immunity was examined, the DNAse activity alone was found be protective except under conditions of excess viral genome copy numbers where Csm6 RNase activity becomes necessary to provide robust immunity (75). The natural high degree of functional variation amongst type III systems is also highlighted by the prediction that type III-C through III-F systems are predicted to encode Cas10 (Cmr2 or Csm1) proteins that lack the ability to cut DNA or generate cOA signalling molecules (3).

In addition to these functional differences, the regulatory mechanism that govern spatiotemporal control of the activities of type III systems have also shown to be quite different. For example, we show here clear evidence that the *P. furiousus* III-B system utilizes bipartite recognition of target RNA (through crRNA base-pairing and likely protein-mediated PFS interaction) in stimulating the DNase and RNase activities of the crRNP (Figure 6). In contrast, crRNA pairing alone with the target RNA appears to trigger activity for the *S. epidermidis* III-A system (69). Collectively, the high degree of functional variability argues that diverse type III systems must be extensively characterized to understand the full spectrum of modes of action and regulatory mechanisms operating in type III immunity.

In summary, our findings significantly contribute to our understanding of the multiple mechanisms that ensure that Type III CRISPR systems become appropriately activated and then rapidly deactivated to achieve maximum benefit to host cells experiencing infection from invading mobile genetic elements. In future studies, a detailed biochemical and structural analyses of *Pfu* Type III-B crRNPs in the presence and absence of substrate target RNAs and DNAs and with and without Csx1, would provide important insight into specific molecular mechanisms that control the timing and specificity of Type III crRNP-mediated immunity.

## Supplementary Material

gkaa176_Supplemental_File
