# Supplementary material for: Regulation of the RNA and DNA nuclease activities required for Pyrococcus furiosus Type III-B CRISPR–Cas immunity
Source: Nucleic Acids Res. 2020 Mar 21;48(8):4418–34. doi: 10.1093/nar/gkaa176 (PMC7192623; doi:10.1093/nar/gkaa176)
Supplement: gkaa176_Supplemental_File [file gkaa176_supplemental_file.docx]

**Supplementary Material for:**

**Regulation of the RNA and DNA nuclease activities required for *Pyrococcus furiosus* Type III-B CRISPR-Cas Immunity**

Kawanda Foster, Sabine Grüschow, Scott Bailey, Malcolm F. White, and Michael P. Terns


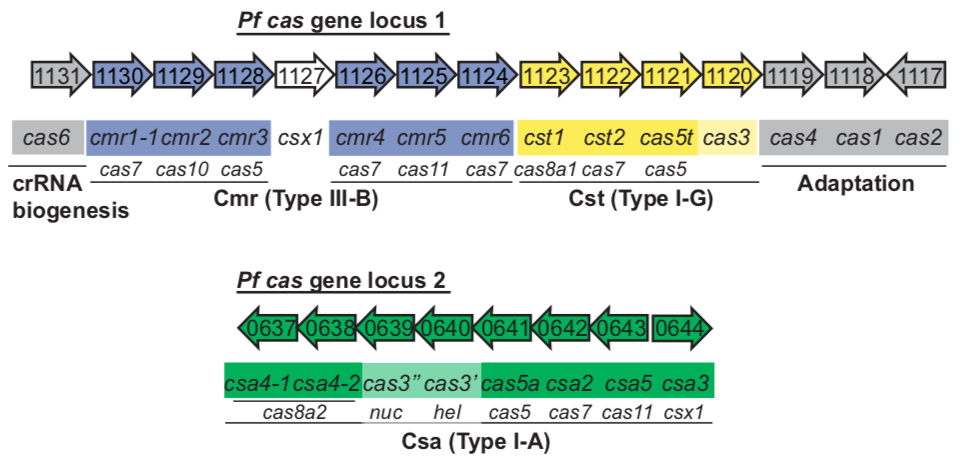


**Supplemental Figure S1.** *Pyrococcus furiosus cas* gene locus organization. The genome organization and annotations of the predicted *cas* genes were adapted from the NCBI database (http://www.ncbi.nlm.nih.gov). *P. furiosus* contains three CRISPR-different Cas systems: Type III-B Cmr (blue), Type I-G Cst (yellow), and Type I-A Csa (green). Specific *cas* gene superfamily designations are indicated below corresponding *csa*, *cst*, and *cmr* genes. Adaptation (gray) and crRNA biogenesis (gray) genes are shared amongst all three systems. Csx1 is not a stable member of the *P. furiosus* Cmr crRNP but the *csx1* gene (white) is often found in association with Type III-B systems and is encoded in between the *cmr3 and cmr4* genes in *Pfu*.

**Supplemental Figure S2.** Target RNAs utilized for *in vitro* assays were created by *in vitro* transcription. Each target RNA contains a different sequence in the PFS region (5’-3’) of the target RNAas as indicated. 100 ng of each RNA was separated by electrophoresis on a 15% denaturing PAGE gel and visualized by ethidium bromide staining.

10 20 30 40 50 60

Pfu MGMRVLVTTWGNPFQWEPITYEYRGIKVKSRNTLPILVKTLEPERILILVADTMANYYDSGKNKPEI

Sis --MKCLFYIAGDVSNYSIVNYELNGQTQNTFFAAHALYNLFKPDKVIALIPDSLVKDNVS------- Sso -MASIVFSTIGNPKGYQKVTYEIDGEKFESNVSVLALRDLLKVDKTVVILGISVADVYNC-------

70 80 90 100 110 EVLPGVG 130

Pfu EEKSFSSYSEVVEDTKERILWHIKEEVIEELREEDPELAKKIENMLKDERITIEVLPGVGVFGNITV

Sis ------DEECYK----NLVINRAKE----------LNFAGMEE---FMNKVEIRKIPNVGIASAIQC Sso ---KYADYRSCK----ECIIQNSKN--------------------DLGISESYVVAPNVY----QKF

140 150 160 IN 180

Pfu EG--------------------EMLDFYYYATYKLAEWLPVQNNLEVYLDLTHGINFMPTFTYRALR

Sis ENGAPKKEKNKEGREVLKRLPYNEKRSPIFIFNAIYAIFKDEACDEYLVDLTHGTNVLVSIGMNVGA Sso KG--------------------KPDHYFTYIYYHSLRILEKEGINEVFIDTTHGINYMGVLAKEAIQ

190 200 210 220 230

Pfu NLLGLLAYL--YNVKFEIVNSEPYPLGVSQEIREDTILHIREIGEGV-VRPRPQYS----PVEG---

Sis LF---NAKF----------YSAPVMGMPGKDSIVNIVE----LT--DVVQATNDSLMIRSSIENLDE Sso LAVSAYAAKSEKEVKVSLYNSDPVGKDVSDTVKLHEIE----AI--K-ISPL--SG-----------

240 250 260

Pfu -----------------------KLYWNAFIS------------SVANGFPLVFASFYPN----IRD

Sis RYFKDYSAKLSRLNPTIFEE-EEKKVLTRVKGT-DVNVVINFLWNIRNGFTVNAVKSMNELKNIINQ Sso ---------LKYVTYQ-ILN-KDKNFFNKIFS-DSVNAIPRFATALDNGLFIYLSEKDSSL--HLKR

270 280 290 300 310 320

Pfu VEDYLNKKLEEFLVGIEVGEREDGKPYVK-REKALDRSFK-----NASKLYYALRVFNTKFQNYPKK Sis LEEDLEKL-KSFYKNWEEHKNFQGETLLVLSDLDSTLKVKDLLI-------------EGN------- Sso LEDDLSK-----------------DPLLTPSENEINVVYKDMKYALSHALFYVISRFSGNV------

330 340 360 370 380

Pfu EVPIEEIMEISKIFESLPRIGIILERQVEWLRNLVYGRLWY------------ENGEQKIKKGLLEI Sis --------------------------------DLEKLNYLLDLYIKASIYDKALSLARELPVAICLN

Sso --------------------------------DLDTLRHYA------------ETYADKVTRAIIEN

390 400 410 420 430 H

Pfu IK------DKKDKRKEAEA--LKKGKTISLAEAAKLTRIFSPSGERIETIESP-NVV**RNFIAH**SGFE Sis KVGGGMFDDKNEKYKHCNE--IV--------------------TSYLRLRYSGLMEFRNTLMHGGLS Sso EV------DKIEKYQMGSERKLL--------------------GEYMKVEGKG-FDKRILYAHGGLP

450 460 470 480

Pfu YNIV---------------------YVKYDRLSDRLYFFYKDKEKAANLAY--EALLYRGEKE- Sis TDMKPNVDKDPNVDKDGNINITPGKIVTKNKIEDFVKRELRN---------YF-DKIVNFLSSA Sso YAGT---------------------YVYKE--KDKVYVTYGD---------KI-DEIERQIG-S

**Figure S3.** Sequence alignment of Csx1 from *P. furiosus (Pfu)*, *S. islandicus* (*Sis*) and *S. solfataricus* (*Sso*). The CARF motif is colored blue. The I169 and N170 are colored red. H436 from the HEPN motif is colored green. The numbering is for *Pfu* Csx1.

**Supplemental Table S1: *Pyrococcus furiosus* Strains**

| **Strain** | **Relevant Characteristics** | **Source** |
| --- | --- | --- |
| Null (TPF20) | ∆*pyr*F ,∆*trp*AB, ∆Cmr (∆*PF1124-PF1130*), ∆Cst (∆*PF1121-1123*) , & ∆Csa (∆*PF0637-0644*) | Elmore et al, 2015 |
| Cmr (TPF15) | ∆*pyr*F ,∆*trp*AB ,∆Cst (∆*PF1121-1123*) & ∆Csa(∆*PF0637-0644*) | Elmore et al, 2015 |
| HD_m_ (TPF63) | ∆*pyr*F ,∆*trp*AB ,∆Cst (∆*PF1121-1123*), ∆Csa(∆*PF0637-0644*), & *cmr2-*H13A/D14A | Elmore et al, 2015 |
| Palm_m_ (TPF27) | ∆*pyr*F ,∆*trp*AB ,∆Cst (∆*PF1121-1123*), ∆Csa(∆*PF0637-0644*), & *cmr2-*D673A/D674A | Elmore et al, 2015 |
| Csx1_∆_ (TPF24) | ∆*pyr*F ,∆*trp*AB ,∆Cst (∆*PF1121-1123*), ∆Csa(∆*PF0637-0644*), & ∆*csx1*(∆PF1127) | Elmore et al, 2015 |
| HD_m_ / Palm_m_ (TPF37) | ∆*pyr*F ,∆*trp*AB ,∆Cst (∆*PF1121-1123*), ∆Csa(∆*PF0637-0644*), & *cmr2-*H13A/D14A*,* D673AD674A | Elmore et al, 2015 |
| Palm_m_ / Csx1_∆_ (TPF119) | ∆*pyr*F ,∆*trp*AB ,∆Cst (∆*PF1121-1123*), ∆Csa(∆*PF0637-0644*), *cmr2-*D673A/D674A, & ∆*csx1*(∆PF1127) | This study |
| HD_m_ / Csx1_∆_ (TPF118) | ∆*pyr*F ,∆*trp*AB ,∆Cst (∆*PF1121-1123*), ∆Csa(∆*PF0637-0644*), *cmr2-*H13A/D14A, & ∆*csx1*(∆PF1127) | This study |
| HD_m_ / Csx1_HEPN_ (TPF128) | ∆*pyr*F ,∆*trp*AB ,∆Cst (∆*PF1121-1123*), ∆Csa(∆*PF0637-0644*), *cmr2-*H13A/D14A, & *csx1-*H436A | This study |
| HD_m_ / Csx1_CARF_ (TPF136) | ∆*pyr*F ,∆*trp*AB ,∆Cst (∆*PF1121-1123*), ∆Csa(∆*PF0637-0644*), *cmr2-*H13A/D14A, & *csx1-*∆121-127 | This study |
| HD_m_ / Csx1_INQQ_ (TPF138) | ∆*pyr*F ,∆*trp*AB ,∆Cst (∆*PF1121-1123*), ∆Csa(∆*PF0637-0644*), *cmr2-*H13A/D14A, & *csx1-*I169Q/N170Q | This study |
| HD_m_ / Csx1_C_ (TPF131) | ∆*pyr*F ,∆*trp*AB ,∆Cst (∆*PF1121-1123*), ∆Csa(∆*PF0637-0644*), *cmr2-*H13A/D14A, & +*csx1*(+PF1127) | This study |
| Cmr2_c_ / Csx1_∆_ (TPF132) | ∆*pyr*F ,∆*trp*AB ,∆Cst (∆*PF1121-1123*), ∆Csa(∆*PF0637-0644*), +*cmr2 (+PF1129)*, & ∆*csx1*(∆PF1127) | This study |

**Supplemental Table S2: Plasmids**

| **Plasmid** | **Relevant Characteristics** | **Purpose** | **Source** |  |  |  |  |
| --- | --- | --- | --- | --- | --- | --- | --- |
| PyrF-SOE (pJFW18) | AprR general cloning vector with *E.coli* OriT, *Pfu* Pgdh-pyrF cassette, and *Pfu* OriC for replication in *P.furiosus* | *Pfu* strain construction | Farkas et al. 2011 |  |  |  |  |
| No target  (pJE47) | pJFW18 derivative; Tk-csg promoter/Tk-chiA terminator expression cassette | Plasmid silencing assays  (Figures 1, 4) | Elmore et al, 2015 |  |  |  |  |
| Target  (pJE65) | pJE47 derivative; 7.01 spacer, GGG flank, target strand transcribed | Plasmid silencing assays (Figures 1, 4)  IVT Target RNA Production (Figures 2, 4, 6; Table 1) | Elmore et al, 2015 |  |  |  |  |
| Target  (pJE186) | pJE47 derivative; 7.01 spacer, TTT flank, target strand transcribed | IVT Target RNA Production (Figure 6; Table 1) | Elmore et al, 2015 |  |  |  |  |
| Target  (pJE187) | pJE47 derivative; 7.01 spacer, TTC flank, target strand transcribed | IVT Target RNA Production (Figure 6; Table 1) | Elmore et al, 2015 |  |  |  |  |
| Target  (pJE189) | pJE47 derivative; 7.01 spacer, TTG flank, target strand transcribed | IVT Target RNA Production (Figure 6; Table 1) | Elmore et al, 2015 |  |  |  |  |
| Target  (pJE190) | pJE47 derivative; 7.01 spacer, TCT flank, target strand transcribed | IVT Target RNA Production (Figure 6; Table 1) | Elmore et al, 2015 |  |  |  |  |
| Target  (pJE193) | pJE47 derivative; 7.01 spacer, TCG flank, target strand transcribed | IVT Target RNA Production (Figure 6; Table 1) | Elmore et al, 2015 |  |  |  |  |
| Target  (pJE194) | pJE47 derivative; 7.01 spacer, TAT flank, target strand transcribed | IVT Target RNA Production (Figure 6; Table 1) | Elmore et al, 2015 |  |  |  |  |
| Target  (pJE197) | pJE47 derivative; 7.01 spacer, TAG flank, target strand transcribed | IVT Target RNA Production (Figure 6; Table 1) | Elmore et al, 2015 |  |  |  |  |
| Target  (pJE198) | pJE47 derivative; 7.01 spacer, TGT flank, target strand transcribed | IVT Target RNA Production (Figure 6; Table 1) | Elmore et al, 2015 |  |  |  |  |
| Target  (pJE201) | pJE47 derivative; 7.01 spacer, TGG flank, target strand transcribed | IVT Target RNA Production (Figure 6; Table 1) | Elmore et al, 2015 |  |  |  |  |
| Target  (pJE202) | pJE47 derivative; 7.01 spacer, CTT flank, target strand transcribed | IVT Target RNA Production (Figure 6; Table 1) | Elmore et al, 2015 |  |  |  |  |
| Target  (pJE205) | pJE47 derivative; 7.01 spacer, CTG flank, target strand transcribed | IVT Target RNA Production (Figure 6; Table 1) | Elmore et al, 2015 |  |  |  |  |
| Target  (pJE207) | pJE47 derivative; 7.01 spacer, CCC flank, target strand transcribed | IVT Target RNA Production (Figure 6; Table 1) | Elmore et al, 2015 |  |  |  |  |
| Target  (pJE214) | pJE47 derivative; 7.01 spacer, CGT flank, target strand transcribed | IVT Target RNA Production (Figure 6; Table 1) | Elmore et al, 2015 |  |  |  |  |
| Target  (pJE217) | pJE47 derivative; 7.01 spacer, CGG flank, target strand transcribed | IVT Target RNA Production (Figure 6; Table 1) | Elmore et al, 2015 |  |  |  |  |
| Target  (pJE221) | pJE47 derivative; 7.01 spacer, ATG flank, target strand transcribed | IVT Target RNA Production (Figure 6; Table 1) | Elmore et al, 2015 |  |  |  |  |
| Target  (pJE228) | pJE47 derivative; 7.01 spacer, AAA flank, target strand transcribed | IVT Target RNA Production (Figure 6; Table 1) | Elmore et al, 2015 |  |  |  |  |
| Target  (pJE230) | pJE47 derivative; 7.01 spacer, AGT flank, target strand transcribed | IVT Target RNA Production (Figure 6; Table 1) | Elmore et al, 2015 |  |  |  |  |
| Target  (pJE233) | pJE47 derivative; 7.01 spacer, AGG flank, target strand transcribed | IVT Target RNA Production (Figure 6; Table 1) | Elmore et al, 2015 |  |  |  |  |
| Target  (pJE237) | pJE47 derivative; 7.01 spacer, GTG flank, target strand transcribed | IVT Target RNA Production (Figure 6; Table 1) | Elmore et al, 2015 |  |  |  |  |
| Target  (pJE246) | pJE47 derivative; 7.01 spacer, GGT flank, target strand transcribed | IVT Target RNA Production (Figure 6; Table 1) | Elmore et al, 2015 |  |  |  |  |
| Target  (pJE247) | pJE47 derivative; 7.01 spacer, GGC flank, target strand transcribed | IVT Target RNA Production (Figure 6; Table 1) | Elmore et al, 2015 |  |  |  |  |
| Target  (pJE248) | pJE47 derivative; 7.01 spacer, GGA flank, target strand transcribed | IVT Target RNA Production (Figure 6; Table 1) | Elmore et al, 2015 |  |  |  |  |

**Supplemental Table S3: Oligos**

***In Vitro* Assay Oligos**

| **Oligo** | **Sequence (5’-3’)** | **Purpose** |
| --- | --- | --- |
| 7.01 crRNA | AUUGAAAGUUGUAGUAUGCGGUCCUUGCGGCUGAGAGCACUUCAG | crRNA used for assembling Cmr crRNPs |
| 7.01 Target RNA | CUGAAGUGCUCUCAGCCGCAAGGACCGCAUACUACAAGGG | RNA substrate used for Cmr crRNP and Csx1 cleavage assays |
| DNA Target 1 | AGCGACAGAGTTTAGGTATGAATGATTAGATAGAGGTAGTGAAGTGGTGTGAGATGGAAGGAGAGGATAGTAGAAGGGTAGTTAGTATTAGATAGAGGTAAGTGATCCCAGTACGTCGTAGTA | DNA substrate used for Cmr crRNP cleavage assays (annealed to DNA target 2) |
| DNA Target 2 | TACTACGACGTACTGGGATCACTTACCTCTATCTAATACTAACTACCCTTCTACTATCCTCTCCTTCCATCTCACACCACTTCACTACCTCTATCTAATCATTCATACCTAAACTCTGTCGCT | DNA substrate used for Cmr crRNP cleavage assays (annealed to DNA target 1) |

**SOE-PCR Construct Primers**

| **Primer** | **Sequence (5’-3’)** | **Purpose** |
| --- | --- | --- |
| Pgdh_PyrF_F | GATTGAAAATGGAGTGAGCTGAG | Used with pJFW18 to amplify the PyrF marker for all constructs |
| Pgdh_PyrF_R | TTATCTTGAGCTCCATTCTTTCACC | Used with pJFW18 to amplify the PyrF marker for all constructs |
| ∆Csx1_1 | GGCAGAATTTACCCCCTTCC | Used to add a Csx1 deletion into existing Palm_m_ or HD_m_ strains |
| ∆Csx1_2 | CTCAGCTCACTCCATTTTCAATCTCATTCCCATATCCCTCCTAAAGC | Used to add a Csx1 deletion into existing Palm_m_ or HD_m_ strains |
| ∆Csx1_5 | GGTGAAAGAATGGAGCTCAAGATAATCCCACAATAGGGAAAGTTGG | Used to add a Csx1 deletion into existing Palm_m_ or HD_m_ strains |
| ∆Csx1_6 | TCATTCCCATATCCCTCCTAAAGC | Used to add a Csx1 deletion into existing Palm_m_ or HD_m_ strains |
| ∆Csx1_7 | GCTTTAGGAGGGATATGGGAATGACTGCAAATCTCGCTTATGAAG | Used to add a Csx1 deletion into existing Palm_m_ or HD_m_ strains |
| ∆Csx1_8 | CCTTTGCCCTGGGAGTTACA | Used to add a Csx1 deletion into existing Palm_m_ or HD_m_ strains |
| Csx1_H436A_1 | TAAGAAACTTGCTTGGATTGTTG | Used to add a Csx1 H436A mutation into the existing HD_m_ strain |
| Csx1_H436A_2 | CTCAGCTCACTCCATTTTCAATCTGCTATAAAGTTACGAACAACATTTG | Used to add a Csx1 H436A mutation into the existing HD_m_ strain |
| Csx1_H436A_5 | GGTGAAAGAATGGAGCTCAAGATAATTTAGAGAGGCAAGTAGAGTG | Used to add a Csx1 H436A mutation into the existing HD_m_ strain |
| Csx1_H436A_6 | TGCTATAAAGTTACGAACAACATTTG | Used to add a Csx1 H436A mutation into the existing HD_m_ strain |
| Csx1_H436A_7 | CAAATGTTGTTCGTAACTTTATAGCAGCATCTGGATTTGAGTATAACATTGTC | Used to add a Csx1 H436A mutation into the existing HD_m_ strain |
| Csx1_H436A_8 | AGTTCCTGTCTCAGCATTAATT | Used to add a Csx1 H436A mutation into the existing HD_m_ strain |
| Csx1_CARF_1 | CAGGATGCGGAATCAAAGATA | Used to delete residues 121-127 from Csx1 in the existing HD_m_ strain |
| Csx1_CARFdel_2 | CTCAGCTCACTCCATTTTCAATCAATTGTAATTCTTTCATCTTTTAACATATTC | Used to delete residues 121-127 from Csx1 in the existing HD_m_ strain |
| Csx1_CARF_5 | GGTGAAAGAATGGAGCTCAAGATAAAGAAATACCTTGCCAATTCTAG | Used to delete residues 121-127 from Csx1 in the existing HD_m_ strain |
| Csx1_CARFdel_6 | AATTGTAATTCTTTCATCTTTTAACATATTC | Used to delete residues 121-127 from Csx1 in the existing HD_m_ strain |
| Csx1_CARFdel_7 | GAATATGTTAAAAGATGAAAGAATTACAATTGTCTTTGGCAACATTACAGTAG | Used to delete residues 121-127 from Csx1 in the existing HD_m_ strain |
| Csx1_CARF_8 | AAGACCCTTCTTTATTTTCTGTTC | Used to delete residues 121-127 from Csx1 in the existing HD_m_ strain |
| Csx1_CARF_IN_1 | GAAAGCCCTCTTCTATTTCTC | Used to create an I169Q/N170Q mutation in Csx1 in the existing HD_m_ strain |
| Csx1_CARF_IN_2 | CTCAGCTCACTCCATTTTCAATCCCCATGAGTTAGGTCTAAGT | Used to create an I169Q/N170Q mutation in Csx1 in the existing HD_m_ strain |
| Csx1_CARF_IN_5 | GGTGAAAGAATGGAGCTCAAGATAATTCGGAAGTTGTGGAAGATAC | Used to create an I169Q/N170Q mutation in Csx1 in the existing HD_m_ strain |
| Csx1_CARF_IN_6 | CCCATGAGTTAGGTCTAAGT | Used to create an I169Q/N170Q mutation in Csx1 in the existing HD_m_ strain |
| Csx1_CARF_INQ_7 | ACTTAGACCTAACTCATGGGCAGCAGTTCATGCCCACCTTTACTTAC | Used to create an I169Q/N170Q mutation in Csx1 in the existing HD_m_ strain |
| Csx1_CARF_IN_8 | TCCAGAATGTGCTATAAAGTTACG | Used to create an I169Q/N170Q mutation in Csx1 in the existing HD_m_ strain |
| Csx1_NruI_1 | AGGCCTCAAGAATTGTGTTG | Used to complement wildtype Csx1 back into the HD_m_ / Csx1_∆_ strain |
| Csx1_NruI_2 | CTCAGCTCACTCCATTTTCAATCCATATCCCTCCTAAAGCAAG | Used to complement wildtype Csx1 back into the HD_m_ / Csx1_∆_ strain |
| Csx1_NruI_5 | GGTGAAAGAATGGAGCTCAAGATAAATTTCTCAACTCCCACAATAGG | Used to complement wildtype Csx1 back into the HD_m_ / Csx1_∆_ strain |
| Csx1_NruI_6 | CATATCCCTCCTAAAGCAAG | Used to complement wildtype Csx1 back into the HD_m_ / Csx1_∆_ strain |
| Csx1_NruI_7 | CTTGCTTTAGGAGGGATATGGGAATGAGAGTTTTGGTAACTAC | Used to complement wildtype Csx1 back into the HD_m_ / Csx1_∆_ strain |
| Csx1_NruI_8 | CGAATATTTGGATAAAAGCTGGCA | Used to complement wildtype Csx1 back into the HD_m_ / Csx1_∆_ strain |
| Csx1_NruI_9 | TGCCAGCTTTTATCCAAATATTCG**C**GACGTAGAAGATTACCTTAACAAAA | Used to complement wildtype Csx1 back into the HD_m_ / Csx1_∆_ strain |
| Csx1_NruI_10 | TGATTAGGGCCATATCATTATCC | Used to complement wildtype Csx1 back into the HD_m_ / Csx1_∆_ strain |
| Cmr2_BclI_1 | CACAAAGGAAAGAAGCAGAGT | Used to complement wildtype Cmr2 back into the HD_m_ / Csx1_∆_ strain |
| Cmr2_BclI_2 | CTCAGCTCACTCCATTTTCAATCTCTCTTTGATGTTAACCACTCCAA | Used to complement wildtype Cmr2 back into the HD_m_ / Csx1_∆_ strain |
| Cmr2_BclI_5 | GGTGAAAGAATGGAGCTCAAGATAAGGAGGAGAATTAAGAAGTATCAAAG | Used to complement wildtype Cmr2 back into the HD_m_ / Csx1_∆_ strain |
| Cmr2_BclI_6 | TCTCTTTGATGTTAACCACTCCAA | Used to complement wildtype Cmr2 back into the HD_m_ / Csx1_∆_ strain |
| Cmr2_BclI_7 | TTGGAGTGGTTAACATCAAAGAGAAACTTTTTGTATACCTTCATGATCC | Used to complement wildtype Cmr2 back into the HD_m_ / Csx1_∆_ strain |
| Cmr2_BclI_8 | ATCAAACACATGGGACATAATGG | Used to complement wildtype Cmr2 back into the HD_m_ / Csx1_∆_ strain |
| Cmr2_BclI_9 | CCATTATGTCCCATGTGTTTGAT**C**AAAAGGTATTATCCAGTGTGGATTAG | Used to complement wildtype Cmr2 back into the HD_m_ / Csx1_∆_ strain |
| Cmr2_BclI_10 | GTTCTTGTACTATTTCTCCAACTTTC | Used to complement wildtype Cmr2 back into the HD_m_ / Csx1_∆_ strain |

***P. furiosus* Strain Screening Oligos**

| **Primer** | **Sequence (5’-3’)** | **Mutation** |
| --- | --- | --- |
| ∆Csx1_seq_For | GTGTTGGAGTGGGTGAGGAG | Csx1 deletion |
| ∆Csx1_seq_Rev | TCTGGAGATATTTGCCGTTAATC | Csx1 deletion |
| Csx1_H436A_Seq_F | GGTTGCCAGTTCAGAACAAT | Csx1-H436A |
| Csx1_H436A_Seq_R | AGGGCCATATCATTATCCTTTG | Csx1-H436A |
| Csx1_CARF_SeqF | AGCTTTCTAGAGAGAAGAAAGTA | Csx1 ∆121-127 |
| Csx1_CARF_SeqR | AAATATTCTTGTAAGCTTTGCAGC | Csx1 ∆121-127 |
| Csx1_CARF_IN_SeqF | TCATCATATGAGTTCTTAACGTTAG | Csx1- I169Q/N170Q |
| Csx1_CARF_IN_SeqR | TTCATAAGCGAGATTTGCAGCT | Csx1- I169Q/N170Q |
| Csx1_CARF_IN_SeqI | GTCTTTGGCAACATTACAGTAG | Csx1- I169Q/N170Q |
| Csx1_NruI_SeqF | ATGCAGGAAGTGAAAGTGTG | Csx1 complementation |
| Csx1_NruI_SeqInt | TTCACAAGAAATAAGGGAGGAC | Csx1 complementation |
| Csx1_NruI_SeqR | GTCACCTCTTTAAGAGTAACTTTG | Csx1 complementation |
| Cmr2_BclI_SeqF | ATGGTGGTTCAGAGCTTTG | Cmr2 complementation |
| Cmr2_BclI_SeqInt | GGAGAGAGAAAAGTTACAGAAG | Cmr2 complementation |
| Cmr2_BclI_SeqR | TGGCTTCTTTTCATGCATATCC | Cmr2 complementation |

**Csx1 *in vitro* Mutagenesis Oligos**

| **Primer** | **Sequence (5’-3’)** | **Mutation** |
| --- | --- | --- |
| Csx1_CARFdel_F | GTCTTTGGCAACATTACAGTAGAG | Csx1 ∆121-127 |
| Csx1_CARFdel_R | AATTGTAATTCTTTCATCTTTTAACATATTCTC | Csx1 ∆121-127 |
| Csx1_ 169A170A _F | GTTTACTTAGACCTAACTCATGGGGCTGCTTTCATGCCCACCTTTACTTAC | Csx1- I169A/N170A |
| Csx1_ 169A170A _R | GTAAGTAAAGGTGGGCATGAAAGCAGCCCCATGAGTTAGGTCTAAGT | Csx1- I169A/N170A |
| Csx1_ 169Q170Q _F: | GTTTACTTAGACCTAACTCATGGGCAGCAGTTCATGCCCACCTTTACTTAC | Csx1- I169Q/N170Q |
| Csx1_ 169Q170Q _R: | GTAAGTAAAGGTGGGCATGAACTGCTGCCCATGAGTTAGGTCTAAGT | Csx1- I169Q/N170Q |
| Csx1_ CARFSeq _Fwd | GCTGATACAATGGCCAACT | Csx1 ∆121-127 or  Csx1- I169A/N170A or Csx1- I169Q/N170Q |
| Csx1_ CARFSeq _Rev | GAATTGTGTCCTCCCTTATTTC | Csx1 ∆121-127 or  Csx1- I169A/N170A or Csx1- I169Q/N170Q |

**IVT Template PCR Primers**

| **Primer** | **Sequence (5’-3’)** |
| --- | --- |
| pJE47_IVT_T7_F | TAATACGACTCACTATAGGGAGACAACACTTAGTAGGGGCTA |
| pJE47_IVT_R | GCTTCCTTAGCTGTTTCTCCA |

**References**

Farkas J, Chung D, DeBarry M, Adams MW, Westpheling J. 2011. Defining components of the chromosomal origin of replication of the hyperthermophilic archaeon Pyrococcus furiosus needed for construction of a stable replicating shuttle vector. *Applied and environmental microbiology* 77: 6343-6349.

Lipscomb GL, Stirrett K, Schut GJ, Yang F, Jenney FE, Jr., Scott RA, Adams MW, Westpheling J. 2011. Natural competence in the hyperthermophilic archaeon Pyrococcus furiosus facilitates genetic manipulation: construction of markerless deletions of genes encoding the two cytoplasmic hydrogenases. *Applied and environmental microbiology* 77: 2232-2238.

Elmore, J. R., Sheppard, N. F., Ramia, N., Deighan, T., Li, H., Terns, R. M., & Terns, M. P. 2016. Bipartite recognition of target RNAs activates DNA cleavage by the Type III-B CRISPR-Cas system. *Genes & development*, *30*(4): 447–459.
